# Preparation and performance features of wristband samplers and considerations for chemical exposure assessment

**DOI:** 10.1038/jes.2017.9

**Published:** 2017-07-26

**Authors:** Kim A Anderson, Gary L Points, Carey E Donald, Holly M Dixon, Richard P Scott, Glenn Wilson, Lane G Tidwell, Peter D Hoffman, Julie B Herbstman, Steven G O'Connell

**Affiliations:** 1 Department of Environmental and Molecular Toxicology, Oregon State University, Corvallis, Oregon, USA; 2 Columbia Center for Children’s Environmental Health, Department of Environmental Health Sciences, Mailman School of Public Health, Columbia University, New York, New York, USA

**Keywords:** flame retardants, PCBs, pesticides, personal exposure, polycyclic aromatic hydrocarbons, volatile organic compounds

## Abstract

Wristbands are increasingly used for assessing personal chemical exposures. Unlike some exposure assessment tools, guidelines for wristbands, such as preparation, applicable chemicals, and transport and storage logistics, are lacking. We tested the wristband’s capacity to capture and retain 148 chemicals including polychlorinated biphenyls (PCBs), pesticides, flame retardants, polycyclic aromatic hydrocarbons (PAHs), and volatile organic chemicals (VOCs). The chemicals span a wide range of physical–chemical properties, with log octanol–air partitioning coefficients from 2.1 to 13.7. All chemicals were quantitatively and precisely recovered from initial exposures, averaging 102% recovery with relative SD ≤21%. In simulated transport conditions at +30 °C, SVOCs were stable up to 1 month (average: 104%) and VOC levels were unchanged (average: 99%) for 7 days. During long-term storage at −20 °C up to 3 (VOCs) or 6 months (SVOCs), all chemical levels were stable from chemical degradation or diffusional losses, averaging 110%. Applying a paired wristband/active sampler study with human participants, the first estimates of wristband–air partitioning coefficients for PAHs are presented to aid in environmental air concentration estimates. Extrapolation of these stability results to other chemicals within the same physical–chemical parameters is expected to yield similar results. As we better define wristband characteristics, wristbands can be better integrated in exposure science and epidemiological studies.

## Introduction

### Chemical Exposure on Health

Accurately assessing personal exposure to environmental toxicants is central to the challenge of linking chemical exposure and health. For example, exposure to polycyclic aromatic hydrocarbons (PAHs) has been associated with obesity, cancer, decreased intelligence quotient, and respiratory distress,^1, 2, 3^ whereas exposures to flame retardants has been associated with cardiotoxicity, reduced hormone levels, and neurotoxicity.^4, 5, 6^ Yet, for most environmental chemicals, there is little information about the frequency and magnitude of personal exposure.^7, 8^ The lack of low-cost, easy-to-use personal sampling technology currently hinders several fields of research, including epidemiology, exposure science, toxicology, and disaster assessment.

### Existing Technologies

Many types of active air sampling devices have been developed to assess chemical exposure, but tend to be costly and complicated to operate.^9^ Fixed stationary active air samplers are common, but may increase uncertainty when estimating individual exposure to pollutants as compared with personal samplers.^10, 11, 12, 13^ To address the need to measure personal chemical exposure, studies have used active air monitoring backpacks^14, 15, 16, 17^ or active samplers on lapels.^18^ Active backpacks include a battery pack and pump that continuously sample air, but the requirement to carry a backpack can be a burden for study participants.^14^ Other concerns include pump noise and weight that can affect compliance,^14, 19, 20^ equipment malfunction during deployment, and cost to the researcher. Active backpack equipment costs ∼$3000 and calibration equipment can cost over $2500. There may be additional costs for pump refurbishment, replacement batteries, and calibration certifications. Although there can be tradeoffs between cost and quality for some approaches, there may be other sampling techniques, such as passive sampling, to measure chemical exposure that provide a quality compromise.

Biological samples such as blood or urine are favored by researchers trying to measure the totality of personal environmental chemical exposures.^7, 8, 19^ However, it is challenging to identify biomarkers with robust specificity and sensitivity.^20, 21^ In addition, it is difficult to accurately control for inter- and intra-individual variability because of factors influencing metabolic capacity, chemical toxicokinetics, and exposure event timing.^22, 23, 24^ Furthermore, biomarker analysis for non-persistent chemicals with short biologic half-lives can lead to exposure misclassification.^24, 25^ Biomarkers also do not indicate the source of chemical exposure, but rather integrate all exposure routes such as inhalation, ingestion, and dermal contact.^26^ There are relatively few measurable biomarkers that can be directly linked to exposures. Policy decisions may be informed by biomarkers, but they cannot be used to regulate. Biological sample collection, such as phlebotomy,^25^ can also be invasive and may yield low participation. External measures are needed to characterize and quantify chemicals in an individual’s environment.

### Passive Sampling

For over two decades, passive sampling devices have been used to sample the bioavailable fraction of organic contaminants in numerous environmental media.^27, 28, 29, 30^ Passive samplers have been used to sample non-polar and semipolar chemicals in air,^31, 32, 33^ water,^34, 35, 36^ and sediments.^37, 38^ Passive sampling badges and tubes have been previously used to assess personal exposure to some volatile organic chemicals (VOCs).^39, 40^ Both SVOCs and VOCs occur in air and diffuse into the lipophilic membrane of passive samplers. Concentrations increase until equilibrium is reached with the surrounding sample matrix.^27^ Chemicals absorb into the polymer and permeate throughout the polymer during deployment.^27^ Chemicals in the lipophilic polymer represent the *unbound* fraction of contaminants, making passive samplers a biologically relevant surrogate of chemical exposure.^41, 42, 43, 44^

### History and Characteristics of Wristbands

A recent advancement in passive sampling devices is the invention of the personal wristband chemical monitor, derived from commercially available silicone wristbands.^33^ Silicone wristbands have been used increasingly to provide personal exposure assessments, and are often partnered with demographic data assessed by questionnaires to infer lifestyles and behaviors that are associated with chemical concentrations.^45, 46, 47, 48^ To date, wristbands have been used to measure exposure to PAHs with occupationally exposed roofers,^33^ flame retardants with preschool children,^45^ and pesticides with farmers in developing countries.^47, 48^

Because accurate measurement of chemical exposure is a critical component for estimating health effects, wristbands can complement current methods for personal chemical exposure. For example, wristbands have been paired with conventional methods in two separate studies. Hammel et al.^46^ compared wristbands with hand wipes and urine samples for organophosphate flame retardants (OPFRs), and found more significant correlations between OPFRs in wristbands and corresponding urinary metabolites than by using hand wipes. Similarly in a manuscript in preparation, Dixon et al.compared wristbands with air monitoring backpacks and urine samples for PAHs, and found more significant correlations between PAHs in wristbands and corresponding urinary metabolites than between backpack and urine samples. In both these studies, Wristbands correlate well with metabolites in urine samples, thus demonstrating the utility of wristbands as a biologically relevant exposure tool.

### Establishing Wristband Practices and User Guidelines

As the use of wristbands increases, it is critical to establish a framework for methods and applications, especially as there are alternative ways to prepare, transport, store, and extract chemicals from these samplers. Some sampling technologies are delicate or susceptible to compromise during transport or storage, or their stability and robustness is not well characterized, leading to increased uncertainty for users.^49^ There is a need to provide practical guidelines for preparation, application, transport, and storage of wristbands. It is beneficial to know the conditions needed to retain quality data while not using unnecessary, burdensome protocols. In addition, once samples reach the laboratory, it is not always possible to analyze the samples immediately. Essential information on the criteria for transport and storage of wristbands will increase the utility of this technology for exposure science, epidemiology, and toxicology studies.

The objectives of this study are: (1) to demonstrate that when the wristband is properly prepared it provides a simple, chemically clean foundation for quantitative organic chemical monitoring; (2) to assess the transport and storage stability of captured chemicals in the wristband, and (3) to estimate wristband–air partitioning coefficients, *K*_sa_, for a commonly studied chemical class, PAHs. Together, these aims provide practical guidelines for future wristband users in study design, field deployment, and laboratory analysis.

## Materials and methods

### Standards, Solvents, and Reagents

Solvents were purchased from Fisher Scientific (Pittsburgh, PA, USA). Ethyl acetate was Optima and *n*-hexane was GC-Resolv grade. Analytical grade standards, purity ≥98%, were obtained from Accustandard (New Haven, CT, USA) and selected to represent a range of physicochemical properties. A complete list of chemicals, CAS numbers, and physicochemical parameters are provided in Supplementary Table S1. Internal standards and extraction surrogates are detailed in Supplementary Table S2. Polytetrafluoroethylene (PTFE) transport/storage bags and closures were purchased from Welch Fluorocarbon (Dover, NH, USA).

### Wristband Sampler Conditioning and Preparation

Before use, silicone wristbands (width: 1.3 cm; inner diameter: 5.8 cm, https://24hourwristbands.com, Houston, TX, USA) were baked at 300 °C for 180 min, under vacuum at 0.1 Torr (Vacuum Oven, Blue-M, model no. POM18VC-2, with Welch Duo-seal pump, model no. 1405). The vacuum oven was flushed with 99.99% nitrogen at 15, 30, 45, 60, 90, 120, and 180 min intervals during baking. An in-line liquid nitrogen cold trap was used between the vacuum oven and pump to prevent volatilized impurities from damaging the pump. Wristbands were then stored in sealed metal containers at 4 °C. Air-tight PTFE bags are used for storage before and after deployment.

### Wristband Sampler Analysis

For deployed wristbands used in the partitioning study, wristbands were cleaned with two sequential rinses of 18 MΩ·cm water and one isopropanol rinse to remove superficial fouling or particles. To illustrate the effectiveness of this cleaning process, microscopic images were obtained (Supplementary Figure S1). After deployment, cleaned wristbands were stored in amber glass jars at −20 °C until extraction.

SVOCs were recovered from wristbands with two 100 ml rounds of ethyl acetate extraction at ambient temperature as previously described.^33^ The ethyl acetate was combined and reduced to nominally 1 ml with nitrogen evaporators (Turbo-Vap L, Biotage, Charlotte, NC, USA and N-EVAP 111, Organomation Associates, Berlin, MA, USA). For VOC analyses, wristbands were thermally extracted directly as described below in ‘Volatile organic chemicals (VOCs)’ section.

### Wristband Capture and Transport and Storage Stability

**Figure 1:**
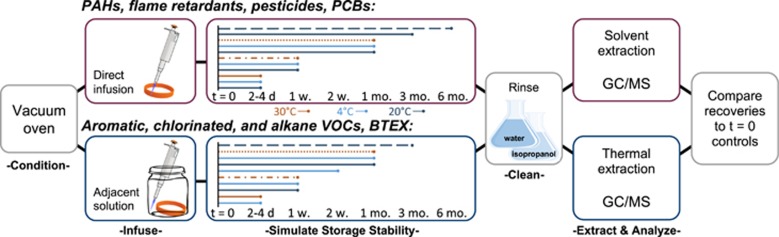
Recovery, transport, and storage stability experimental design. Dashed blue line represents long-term storage, dotted orange line represents accelerated storage stability, and dash–dot line represents transport stability. The cleaning step was only used for wristbands deployed outside the laboratory.

To simulate capture and stability of chemicals within the wristband samplers, 112 wristbands were analyzed. Stability in this context includes degradation of chemical(s) within the wristband or loss(es) through diffusion processes. Wristbands were infused with 148 chemicals using one of two methods: a direct application method of pipetting standards onto the wristbands was used for SVOCs, and a vapor infusion method where gas vapors were allowed to equilibrate with wristbands in a closed glass vessel was used for VOCs. Both methods are further detailed in the Supplementary Information. After infusion, four wristbands were extracted, representing the initial concentrations (*t*=0). Two wristband extracts from separate treatment groups were compromised and thus excluded from subsequent analyses as indicated by *n*=3 in Supplementary Table S6. All other wristbands were stored in clean, air-tight PTFE bags under various temperatures and time point scenarios depicted in Figure 1. Replication consisted of four wristbands extracted for each scenario. Field transport simulations were 2–4 days and 1 week following storage at −20 °C, 4 °C, and +30 °C, and an additional 14 days for VOCS at 4 °C. Long-term storage stability of chemicals in wristbands was evaluated at −20 °C, and included 3 months (82–88 days) for VOCs and 6 months (182 days) for SVOCs.

Accelerated storage stability studies are generally performed at higher temperatures to estimate degradation rates at longer times.^50^ The degradation rate based on accelerated stability studies for the pharmaceutical industry estimates that many reaction rates increase by a factor of two for every 10 °C increase.^50^ Our recommended storage, −20 °C, was used as the basis for calculation, and the accelerated storage stability studies were performed at +30 °C for 1 month. Based on the accelerated study that is 50 °C higher (2^5^) for 28 days (28 × 2^5^=896), the 28-day +30 °C data may roughly estimate chemical stability at ~900 days at −20 °C.

### Instrumental Analysis

Instrumental analysis for the chemicals was performed with five different established analytical methods. For each analytical method additional details are provided in the Supplementary Information, including Supplementary Table S2.

#### Polycyclic aromatic hydrocarbons (PAHs)

A total of 50 alkylated and unsubstituted PAHs were measured with a modified GC-MS/MS with an Agilent 7890B gas chromatograph (Santa Clara, CA, USA) interfaced with an Agilent 7000C mass spectrometer equipped with a triple-axis detector gas chromatograph coupled to tandem mass spectrometer (GC-MS/MS) as detailed elsewhere.^51^ PAHs included 2-, 3-, 4-, 5-, and 6-ring PAHs.

#### Flame retardants

Triphenylphosphate and seven polybrominated diethyl ether (PBDE) flame retardants were analyzed with an Agilent 7890A gas chromatograph coupled to a 5975C mass spectrometer under electron impact ionization (70 eV). The analytical conditions are further detailed in Kile et al.^45^ and Supplementary Information.

#### Pesticides

A total of 47 pesticides or pesticide degradation products were measured: 27 are classified as insecticides, 7 herbicides, 7 fungicides, and 6 pesticide degradation products. The method reflects our interest in insecticides for their potential human health impacts and includes 21 organochlorine, 3 organophosphate, and 3 pyrethroid insecticides. Pesticides were analyzed on an Agilent 6890N GC with dual 7683 injectors, dual columns, DB-XLB and DB-17MS columns (Agilent), and dual microelectron capture detectors (*μ*-ECD).^47^

#### Polychlorinated biphenyls (PCBs)

Thirteen PCBs including tetra-, penta-, hexa-, and hepta-chlorinated PCBs were measured with an Agilent 7890A gas chromatograph coupled to a 5975C mass spectrometer under electron impact ionization (70 eV).

#### Volatile organic chemicals (VOCs)

A total of 31 alkanes, aromatic and halogen substituted aromatic hydrocarbons were extracted directly with a Markes International M-CTE250I microchamber thermal extractor onto Tenex TA Carbograph 5TD thermal desorption tubes (Markes International, Cincinnati, OH, USA), conditions as detailed in Supplementary Table S3. Sorbed tubes were transferred to the Markes International Series 2 Ultra TC auto-sampler interfaced through a Series 2 Unity with an Agilent 7890A gas chromatograph and mass spectrometer. Instrument conditions are detailed in Supplementary Table S2.

All target chemicals were quantified by the relative response of the internal standard to target chemicals in a 4–9-point calibration curve (*R*^2^ >0.97, Supplementary Information and Supplementary Table S2).

### Paired Wristband and PUF Active Air Monitoring Samplers

Concentrations in the paired study are used to calculate *K*_sa_, a partition coefficient between wristbands and air. Wristbands (prepared as described in O’Connell et al.^33^) and air monitoring backpacks (as described previously in Perera et al.^52^) were deployed simultaneously for 48 h on 22 women in an epidemiological birth cohort at the Columbia Center for Children’s Environmental Health in New York City. Informed consent was obtained in agreement with the Columbia University Institutional Review Board (IRB), the IRB of record. Further details of the air monitoring samplers and limits of detection and quality control are provided in Supplementary Information and Supplementary Table S4. Briefly, a sampler holder (URG-200-25A, URG, Chapel Hill, NC, USA) with a precleaned polyurethane foam (PUF) plug, which collects gas-phase organic molecules, was attached over the shoulder on a backpack strap. Twelve PAH concentrations were above the limit of detections in both the wristband and PUF and were used in subsequent analysis.

Informed consent was obtained in agreement with the Columbia University Institutional Review Board (IRB), the IRB of record.

### Statistical Analysis

Treatment recoveries were scaled as a percentage of the mean control (*t*=0) treatments. For the 148 chemicals, there are 1450 time and temperature scenarios. Mean percent recoveries were compared with *t*=0 control treatments with Dunnett’s tests. Each family of Dunnett’s tests, defined as all comparisons for one chemical, controls for false discovery rates from multiple comparisons, is robust to non-normality, and is appropriate for our sample sizes.^53^ In the paired study, linear regression was used to predict *K*_sa_ from the *K*_oa_ of PAHs at equilibrium. Variance among PAHs in the *K*_sa_ model was not different (Levene’s test *P-*value=0.90). Statistical analyses were performed with JMP Pro 12.0.1. Significance was set at *α*=0.05. Standard deviation from pilot data indicated that treatment groups of *n*=3 were adequate, and an additional replicate was included (*n*=4) in case of sample loss.

### QC Samples

To ensure data quality, over 30% of the total samples analyzed were for quality control (QC) purposes. Blank wristband samples were collected during wristband conditioning and cleaning steps. Solvent extraction blanks were collected by performing the extraction process without wristbands. A mix of surrogate standards were added to all wristbands before extraction to quantify chemical recoveries during the extraction process and averaged 101% across methods (median=98%). To account for any instrument background responses, injections of *n*-hexane solvent were included in all analytical batches. To monitor instrument performance, continuing calibration verification standards (CCVs) were analyzed before and after analytical batches. CCVs consist of a solution with known concentrations for all target chemicals and all were within 20% of the true value for at least 80% of the chemicals, indicating that instrument performance remained consistent throughout the analyses. Additional QC pertaining to the paired wristband and active PUF samples is detailed in the Supplementary Information.

## Results

### Chemically Clean Foundation for Quantitative Organic Chemical Monitoring

**Figure 2:**
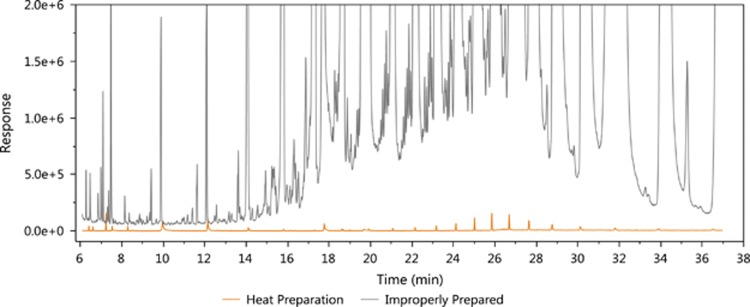
Conditioned wristband in orange shown in the total ion chromatogram (TIC) below, compared with an improperly prepared wristband (gray trace). Note that a wristband without any cleaning would be even further off scale.

After iterative rounds of optimization, we were able to remove oligomers and other chemical contaminants by baking the wristbands under vacuum. We monitored the total mass reduction, the total ion chromatogram (TIC) shown in Figure 2, as well as strength and elasticity properties (Supplementary Figures S2 and S3). For weight reduction, an average of 4% (SD 1.3%, *n*=60) was measured after the method was finalized (Supplementary Figure S3c). By examining the TIC (Figure 2), there is graphical evidence that significant amounts of oligomers were removed. Prepared wristbands were analyzed in five quantitative methods, and none of the 148 target chemicals were above detection limit for any wristband (*n*=6). Cleaned and conditioned wristbands were also screened in a 1400 chemical automated mass spectral deconvolution identification system with deconvolution reporting software, further described in Supplementary Information (*n*=6). Using the 1400 analyte method, only 4 chemicals were detected: di-*n*-butylphthalate, dicyclohexyl phthalate, bis(2-ethyhexyl)phthalate, and methylphenol. In all cases, the 4 chemicals detected after cleaning were present at low levels (<500 p.p.b.), and would need to be subtracted from concentrations measured in deployed wristband samples if quantitated and reported in additional studies. Elasticity, strength, and color were adequate for use, and further test details of these parameters are provided in Supplementary Information and Supplementary Table S5. Microscopic images of the wristband surface after deployment indicate that there are few particulates on the wristband surface before cleaning (Supplementary Figure S1a and b), and even less after cleaning (Supplementary Figure S1c and d).

### Recovery, Transport, and Storage Stability

**Figure 3:**
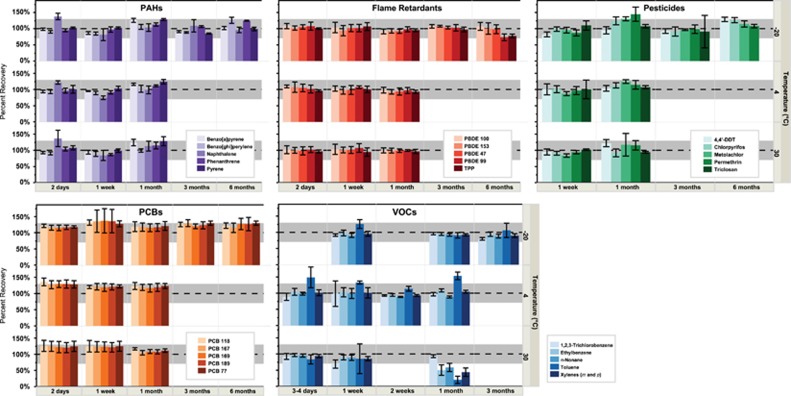
Select chemicals from five broad classes of chemicals. Sets include stability at 4, 7, 28, 82, and 182 (or 183) days (*x* axis) and −20 °C, 4 °C, and +30 °C are organized as three groups on the *y* axis. The percentage recovery for each chemical is given by the *y* axis (scale on left). Stability out to 183 days is reported for −20 °C for all SVOC chemical classes. Recoveries at 2–4, 7, and 28 days at both 4 °C and +30 °C are given for all chemical classes. Each bar represents *n*=4 for each chemical at each time and temperature, with error bars representing standard error. The gray bar across each graph bounds ±30% of the true value (100%). Within pesticides are representative chemicals from insecticides, herbicides, and fungicides. Within the flame retardants are both an organophosphate and PBDEs. PCBs include tetra-to hepta-chlorinated congeners, and PAHs include 2–6-ring chemicals. VOCs include aromatic, chlorinated, and alkane chemicals.

A total of 148 chemicals were recovered from the initial infusions (Table 1), with <21% relative standard deviation (RSD). For all time points and chemical classes, the majority of stability estimates are within 30% of starting concentrations (Figure 3). For the 148 chemicals, there were 1450 time and temperature scenarios in total. Of these, 88 scenarios (6%) were both significantly different and less than *t*=0 controls. However, not all significant differences are meaningful in the context of storage stability. For example, of 88 scenarios, only 29 were both significant and below 70% of initial recoveries. These 29 instances include, in particular, 17 VOCs from the 1-month time point at +30 °C that exhibited low concentrations (average 66% for VOCs). Of the remaining significantly decreased time points (*n*=58), they represent sporadic decreases that are inconsistent with subsequent time points. For example, at −20 °C, 2,3-dimethylanthracene is significantly different from starting concentrations, but is still recovered on average ∼78%. This suggests that some variability is inherent in the infusion process or analytical methods, and does not specifically reflect chemical stability in the wristbands. Furthermore, ∼18% of the total 1450 scenarios were statistically different and *greater* than *t*=0 control, and are reasonably estimated to be an artifact of variability in the infusion process and/or analytical method for those particular compounds. Supplementary Table S6 gives percent recoveries for all time and temperature scenarios. Overall, given potential variability from the infusion, extraction, retention in the polymer, and the analytical method, relative SDs averaged only 9% (median 7%, range <0.1 to 59%) illustrating good precision for most chemicals across a wide breadth of compound diversity.

### Wristband–Air Partition Coefficient Estimates

**Figure 4:**
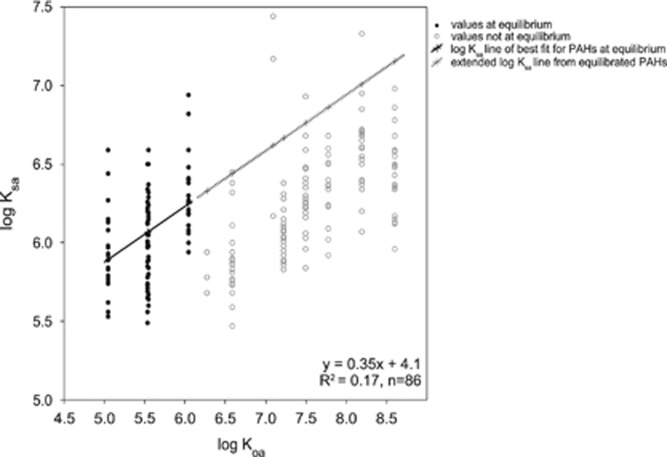
Linear regression plot of log Koa and estimated log Ksa for 12 PAHs in participants who wore paired wristbands and active air-monitoring backpacks. Values in gray circles were generally expected to fall below the line of best fit since they were not at equilibrium at the end of deployment. PAH, polycyclic aromatic hydrocarbon.

The paired wristband and active air monitoring backpack study provide a relationship between calculated log *K*_sa_ (a silicone wristband–air partition coefficient) and log *K*_oa_ for the measured PAHs (wristband and PUF data presented in Supplementary Information). The concentrations of 4 PAHs, naphthalene, 2-methylnaphthalene, 1-methylnaphthalene, and acenaphthene, are estimated to be at equilibrium with air. When wristbands were worn for both 24 h and 7 days by the same individual, it was demonstrated that these 4 lower molecular weight PAHs reached equilibrium after 24 h because concentrations of these 4 PAHs in the wristbands were equal whether worn for 24 h or 7 days (data not shown). Over the 48 h of exposures in the paired wristband and PUF study, the 4 equilibrated PAHs are related to log *K*_oa_ (Eq. (1), Figure 4) with:


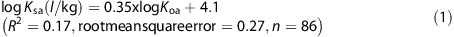


The other 8 PAHs not at equilibrium primarily fall, as expected, below the predicted fit (Figure 4). Using the equation from the 4 PAHs at equilibrium, the estimated *K*_sa_ for all 12 PAHs are calculated (Table 2).

## Discussion

### Properly Conditioned and Prepared Wristbands, a Chemically Clean Tool for Chemical Monitoring

Removal of oligomers and other chemical contaminants that have adsorbed into the wristband is necessary before use because these chemicals adversely affect analytical sensitivity and overall analytical instrument operation. Without proper wristband conditioning, sensitivity is decreased, and the instrument requires excessive maintenance. Higher temperatures or lack of vacuum generally resulted in loss of elasticity or color. However, loss of color has no effect on analytical performance. Although solvent cleaning has been successfully used,^33^ it is more expensive using 42 liters of solvents per 100 wristbands, entails more staff interaction time with the solvent exchanges, and requires disposal of the solvents. Lack of sufficient cleaning led Hammel et al.^46^ to resort to a three-solvent, solid–liquid extraction, and a florisil solid-phase cleanup step of postdeployed wristbands to achieve adequate data objectives. Vacuum-heat conditioning produced clean wristbands, reduced laboratory expenses, and decreased staff time while providing wristbands that can be extracted with a single solvent at room temperature or directly through thermal extraction.

### Recovery, Transport, and Storage Stability

#### Demonstrate chemicals are captured and quantitatively and precisely recovered

Through previous efforts in our laboratory, we know wristbands sequester a wide variety of chemicals including flame retardants,^45^ PAHs,^33^ pesticides,^47^ and personal care products.^48^ Currently, there are few sensor technologies for measuring individual exposure to a wide range of chemicals. Some examples of existing technologies include: brominated flame retardants, OPFRs, and PAHs by active and passive air samplers,^14, 17, 52, 54, 55, 56^ dust and hand wipe samplers,^55, 57^ and biological samples.^58, 59, 60, 61^ The wristband sampler provides one of the only individual sampling technologies available that has been shown to capture both VOCs and SVOCs ranging 10 orders of magnitude of *K*_oa_.^33, 45, 47, 48, 62^ To our knowledge, this is the first demonstration of individual personal samplers that can be used to analyze PCBs, organochlorine pesticides, organophosphate pesticides, pyrethroid pesticides, PBDEs and triphenylphosphate, PAHs, alky substituted PAHs, consumer and industrial chemicals, as well as BTEX and aromatic, chlorinated, and alkane VOCs. While explicitly testing 148 chemicals, extrapolation of these results to other chemicals within the same physical–chemical boundaries is expected to yield similar results, especially for chemicals within chemical classes like PAHs, PCBs, PBDEs, pyrethroids, and alkanes. For example, although we only explicitly tested a single penta-PCB, we would reasonably expect to recover all penta-PCB congeners.

#### Transport and storage stability of VOCs and SVOCs captured in the wristband

We examined wristband transport and storage stability conditions to determine whether temperature or duration influences the recovery of organic chemicals from the wristband samplers. Chemical stability and potential degradation of VOCs and SVOCs during transport and storage are commonly cited problems in complex environmental matrices.^63^ For instance, epidemiological studies collecting biological samples in the field often require special transport considerations, including freezing (e.g., dry ice or colder) samples immediately upon collection that can further complicate a study design and reduce compliance.^64^ Furthermore, longer-term storage stability data are not always obtained or reported for sensors, biological matrices, or sampling techniques that can contribute to increased uncertainty or comparability between studies. The lack of degradation or loss by diffusion of VOCs and SVOCs even with elevated temperature (e.g., 4 °C or +30 °C) over simulated transport conditions provides many practical advantages with regard to study design and compliance. Storage at +30 °C resulted in some loss of VOCs after 1 month that had average recoveries of 66%. Consequently, we would not recommend keeping wristbands for VOC analysis at +30 °C for longer than 7 days.

An accelerated storage stability study at +30 °C for 1 month found 124 of the 148 chemicals were within the 70% to 130% recovery range, and of the 24 chemicals outside the range, 17 were VOCs. Noteworthy, all 31 VOCs were stable at −20 °C at the longest period tested (3 months) and all 117 SVOCs were above 70% recovery at −20 °C for 6 months. We might hypothesize that at longer time periods (e.g., 900 days) some chemical loss might occur especially for VOCs.

Here we demonstrated with simulated long-term storage at −20 °C for 6 months that 105 out of 117 SVOCs were within 70–130% recovery range. All 12 chemicals that were outside this range were above 130% recovery, generally by only a few percent, suggesting SVOCs are stable in wristbands under these conditions. We also demonstrated that all 31 VOCs are stable at −20 °C for at least 3 months. The stability of VOCs and SVOCs while stored provides many practical advantages over other sensors or matrices. For instance, even water USEPA SVOC and VOCs methods 8270 and 8260 maintains that extractions be completed in 7 and 14 days, respectively. The clean wristband polymer may provide a stabilizing matrix platform for many chemicals as compared with more complex matrices.

### Estimate of Silicone–Air Partitioning Coefficients

Although wristbands concentrations can be compared among participants in a study without further calculations, an additional application would be to estimate air concentrations (ng/m^3^) around the individual. For passive samplers, partition coefficients are developed in order to calculate air concentrations from chemical concentrations in the passive sampler. In the present study, paired wristband passive samplers and low-volume personal active samplers were used to estimate *K*_sa_, a silicone wristband–air partition coefficient, for specific PAHs.^65^ Deriving *K*_sa_ values will help researchers calculate concentrations of chemicals in air (i.e., PAHs) from wristbands.

The linear regression of log *K*_oa_ and estimated log *K*_sa_ for 12 PAHs in the paired sampler study indicates that there is a range of *K*_sa_ values for each PAH. This is expected because of differences in wristband personal environments across the 22 study participants. Despite this variability, a relationship emerges between the PAH log *K*_oa_ and the estimated log *K*_sa_ values (Eq. (1)).^65^ Four PAHs were used because they were in equilibrium by the end of the deployment period. For PAHs that did not reach equilibrium during the study, one would expect the *K*_sa_ to be low because the PAH concentration in the wristband silicone has not yet attained equilibrium. As expected, 8 PAHs not at equilibrium in the wristbands all fall below the line of best fit in Figure 3 because their true *K*_sa_ values cannot be calculated until they reach equilibrium. It is consistent to anticipate that the measured log *K*_sa_ of those 8 PAHs, once at equilibrium, would fall along the line of best fit. Future research is warranted to confirm this relationship, and to determine whether *K*_sa_/*K*_oa_ trends are similar for other chemical classes.

Numerous researchers have determined partition coefficients between passive samplers and air using active sampling equipment with models that include several chemical classes, such as PAHs, PCBs, pesticides, and chlorobenzenes.^27, 65, 66^ In these previous works, linear relationships are drawn between log *K*_sa_ and log *K*_oa_, with low sample size, but *R*^2^>0.8. In contrast, our Eq. (1) is derived from 86 data points across 4 PAHs and 22 individuals, resulting in a low model fit *R*^2^=0.17. Interestingly, when we perform a separate linear regression for each of the 22 individuals, model fits are substantially improved with average *R*^2^=0.59 (range: 0.06–0.92, Supplementary Figure S4). However, by using all 22 individuals to predict *K*_sa_, we incorporate variability among individuals when combined into a single regression. Variation is expected to be greater among the wristbands in this study between individuals as both inhalation and dermal exposure sources can be sampled. Moreover, the localized wrist environment likely differs between individuals from external factors such as clothing worn around the sampler or temperature differences during absorption.

### Study Limitations

One challenge is accurately calculating an environmental (or a personal exposure) concentration of a chemical from what is measured in any passive sampling device. This requires either achieving equilibrium for all chemicals, which is not practical, or by adding labeled chemicals to the wristband before deployment to act as performance reference chemicals (PRCs). The amount of PRC that diffuses out of the wristband during deployment can be used to estimate the status of equilibrium for that chemical. With the use of *K*_sa_, as developed here, and PRCs, the environmental concentration can be estimated. PRCs are used to calculate sample-specific, chemical sampling rates by the wristband. The PRC approach is accepted by the scientific community.^27, 65, 67, 68, 69^ The rate at which PRCs diffuse from wristbands is controlled by conditions of the external environment, the properties of the chemical itself, and characteristics of the sampler, such as wristband mass and thickness. PRCs can be added to wristbands in concentrations that are generally regarded as safe. For example, pyrene is a chemical with a relative potency factor for cancer risk of zero^70^ and could be used as a PRC if used in low ng/g wristband concentrations and approved by IRB before deployment.^70, 71^

Passive sampling produces time-integrated concentrations of chemicals.^28, 29^ This can be either a strength or a weakness of the approach, depending on the goals of the study. It is a strength if the objective is to assess a person’s total individual chemical exposure over a period of time, or to measure an average concentration of a chemical over a given time period.^32, 72^ It is a weakness, however, if the objective is to capture the elevated concentration at a specific moment during a pulse of contamination.

Wristbands used in this paired study to develop *K*_sa_ values were worn on the wrist, and potentially incorporate some dermal along with the gas-phase PAH exposure routes. Additional work is needed to parse out the influence of dermal and gas-phase PAH exposure to silicone–air partition coefficients that likely varies by chemical. In future studies, wristbands may also be worn on clothing such as a lapel, as demonstrated in O’Connell et al.,^33^ when environmental exposure studies are intended to include only measures of inhalation exposures.

## Conclusions

We present a quantitative methodology that can measure volatile and semivolatile chemicals in wristband samplers that would complement current monitoring methods. Many chemicals classes were illustrated spanning a log *K*_oa_ range of 2.1 to 13.7, and it is expected that other chemicals, although not explicitly tested in this study, would have similar stability characteristics. General guidelines for transport in air-tight bags for 1 or 2 weeks, even at +30 °C, are acceptable. Storage at 4 °C or −20 °C for SVOC and −20 °C for VOCs out to 6 and 3 months, respectively, is also acceptable. Based on the accelerated storage stability results, longer storage times at −20 °C will likely also be successful and studies to confirm this are currently underway. We also provide the first estimates of wristband–air partition coefficients for future use in determining environmental concentrations. Wristbands are a non-invasive, easy-to-use candidate technology that measures personal exposure to multiple non-polar and semipolar, volatile and semivolatile organic chemical mixtures.

## Figures and Tables

**Table 1:** Summary data from percent recovery, transport, and stability across 148 chemicals.

*Chemical group*	*Log* K_*oa*_ *range*	*Initial recovery from exposure*			*Transport stability 1 week, +30 °C*			*Accelerated storage stability: 1 month, +30 °C*			*Long-term storage or archive: 3–6 months, −20 °C*		
		*Recovery range (%)*	*Recovery (%) AVG; median*	*RSD (%) Range*	*Stability range (%)*	*Stability (%) AVG; median*	*RSD (%) range*	*Stability range (%)*	*Stability (%) AVG; median*	*RSD (%) range*	*Stability range (%)*	*Stability (%) AVG; median*	*RSD (%) range*
PAHs (50)	5.0–13.7	40–138	101; 103	0–20	72–103	89; 90	2–19	89–150	117; 116	2–23	85–142	107; 104	1–11
Flame retardants (7)	8.5–13.3	103–168	121; 113	3–7	95–108	103; 104	9–15	97–103	100; 100	2–8	74–108	95; 101	7–18
Pesticides (47)	6.8–13.5	66–160	120; 119	3–21	81–155	98; 95	2–21	69–135	116; 120	2–30	105–140	120; 120	4–10
PCBs (13)	8.6–11.7	92–112	96; 95	7–9	121–128	124; 124	10–16	104–118	110; 110	3–8	116–168	130; 130	5–34
VOCs (31)	2.7–5.2	81–185	102; 90	0–20	66–99	85; 88	8–54	20–94	64; 67	4–57	73–107	93; 91	3–21
Total (148)	2.7–13.7	40–168	102; 99	0–21	66–155	95; 93	2–54	20–150	104; 113	2–57	73–168	110; 103	3–34

**Table 2:** The predicted *K*_sa_ values (± 95% prediction interval) for 12 PAHs using Eq. (1).

*PAH*	*log* K_*oa*_	*Predicted log* K_*sa*_ *(l/kg)*
Naphthalene	5.0	5.9±0.3
2-Methylnaphthalene	5.5	6.1±0.3
1-Methylnaphthalene	5.5	6.1±0.3
Acenaphthene	6.0	6.2±0.3
Acenaphthylene	6.3	6.3±0.4
Fluorene	6.6	6.4±0.4
Anthracene	7.1	6.6±0.4
Phenanthrene	7.2	6.7±0.5
2-Methylphenanthrene	7.5	6.8±0.5
1-Methylphenanthrene	7.8	6.9±0.5
Pyrene	8.2	7.0±0.6
Fluoranthene	8.6	7.2±0.6
