# Supplementary material for: Preparation and performance features of wristband samplers and considerations for chemical exposure assessment
Source: J Expo Sci Environ Epidemiol. 2017 Jul 26;27(6):551–9. doi: 10.1038/jes.2017.9 (PMC5658681; doi:10.1038/jes.2017.9)
Supplement: Supplementary Information [file jes20179x1.docx]

**Supplementary Information**

**Preparation and performance features of wristband samplers and considerations for chemical exposure assessment**

Running title: Silicone wristbands and chemical exposures

Kim A. Anderson*****^1^, PhD, Gary Points III^1^, BS, Carey E. Donald^1^, PhD, Holly M. Dixon^1^, BS, Richard P. Scott^1^, BS, Glenn Wilson^1^, BS, Lane Tidwell^1^, PhD, Peter Hoffman^1^, BS, Julie Herbstman^2^, PhD, and Steven G. O’Connell^1^, PhD

^1^Department of Environmental and Molecular Toxicology, Oregon State University, Corvallis, Oregon, USA

^2^Columbia Center for Children’s Environmental Health, Department of Environmental Health Sciences, Mailman School of Public Health, Columbia University, New York, New York, USA

***Correspondence:**

Kim A. Anderson

Oregon State University

Department of Environmental and Molecular Toxicology

1007 Agricultural and Life Sciences Building

Corvallis, Oregon 97331, USA

Telephone: (541) 737-8501

Fax: (541) 737-0497

Email: [kim.anderson@oregonstate.edu](mailto:kim.anderson@oregonstate.edu)

SOURCES OF SUPPORT

This research was supported by the Food Safety and Environmental Stewardship Program at Oregon State University and NIEHS R21 ES024718 and R33 ES024718.

**Supplemental Information**

**Table of Contents**

**Page 3-6 Table S1**. Physicochemical properties and CAS number

**Page 7 Table S2:** Analytical instrument parameters

**Page 8 Table S3.** Analytical parameters for micro-chamber thermal extraction

Wristband infusion additional details

**Page 9-10** Paired wristband and active air monitoring backpack PUF study additional details

**Page 11** **Table S4**. Dates of paired wristband and PUF study

**Page 12 Figure S1:** Microscopic images of a wristband after deployment

**Page 13** Conditioned wristband integrity details

**Page 14 Figure S2 A, B, C:** Mean mass changes, percentage lost after wristband conditioning

**Page 15** **Figure S3 A, B**: Strength and elasticity measurement apparatus

**Table S5 A, B, C**: Measure of strength and elasticity

**Page 16 Figure S4.** Individualized linear regression plots of log K_oa_ and estimated log K_sa_

**Page 17-21**  **Table S6:** Average recovery for each time and temperature scenario

**Page 22 References**

**Table S1.** Physioshemical properties and CAS numbers. For log Koa, bold indicates values from KOAWIN v1.10; other values are experimental. For log K_ow_, bold indicates values from KOWWIN v1.76; other values are experimental.

|  | **Compound Name** | **log K_oa_** | **log K_ow_** | **CAS Number** |
| --- | --- | --- | --- | --- |
| PAHs | 1,2-dimethylnaphthalene | **5.892** | 4.31 | 573-98-8 |
|  | 1,4-dimethylnaphthalene | **6.172** | 4.37 | 571-58-4 |
|  | 1,5-dimethylnaphthalene | **6.224** | 4.38 | 571-61-9 |
|  | 1,6-dimethylnaphthalene | **6.022** | 4.44 | 575-43-9 |
|  | 1,8-dimethylnaphthalene | **6.224** | 4.26 | 569-41-5 |
|  | 1-methylnaphthalene | **5.547** | 3.87 | 90-12-0 |
|  | 1-methylphenanthrene | **7.776** | 5.08 | 832-69-9 |
|  | 1-methylpyrene | **8.907** | **5.48** | 2381-21-7 |
|  | 2,3-dimethylanthracene | **8.033** | **5.44** | 613-06-9 |
|  | 2,6-diethylnaphthalene | **6.585** | **4.30** | 59919-41-4 |
|  | 2,6-dimethylnaphthalene | **5.892** | 4.31 | 581-42-0 |
|  | 2-ethylnaphthalene | **6.038** | 4.38 | 939-27-5 |
|  | 2-methylanthracene | **6.586** | 5.00 | 613-12-7 |
|  | 2-methylnaphthalene | **5.534** | 3.86 | 91-57-6 |
|  | 2-methylphenanthrene | **7.495** | 4.86 | 2531-84-2 |
|  | 3,6-dimethylphenanthrene | **8.033** | **5.44** | 1576-67-6 |
|  | 6-methylchrysene | **9.716** | **6.07** | 1705-85-7 |
|  | 7,12-dimethylbenz[a]anthracene | **9.613** | 5.80 | 57-97-6 |
|  | 9,10-dimethylanthracene | **8.283** | 5.69 | 781-43-1 |
|  | 9-methylanthracene | **7.870** | 5.07 | 779-02-2 |
|  | acenaphthene | **6.044** | 3.92 | 83-32-9 |
|  | acenaphthylene | **6.272** | 3.94 | 208-96-8 |
|  | anthanthrene | **12.311** | 7.04 | 191-26-4 |
|  | anthracene | **7.093** | 4.45 | 120-12-7 |
|  | benz[a]anthracene | **9.069** | 5.76 | 56-55-3 |
|  | benzo[a]pyrene | **10.859** | 6.13 | 50-32-8 |
|  | benzo[b]fluoranthene | **10.351** | 5.78 | 205-99-2 |
|  | benzo[c]fluorene | **8.366** | **5.19** | 205-12-9 |
|  | benzo[e]pyrene | **11.351** | 6.44 | 192-97-2 |
|  | benzo[ghi]perylene | **11.499** | 6.63 | 191-24-2 |
|  | benzo[j]fluoranthene | **10.590** | **6.11** | 205-82-3 |
|  | benzo[k]fluoranthene | **10.732** | 6.11 | 207-08-9 |
|  | chrysene | **9.480** | 5.81 | 218-01-9 |
|  | coronene | **13.702** | 7.64 | 191-07-1 |
|  | cyclopenta[c,d]pyrene | **10.151** | **5.70** | 27208-37-3 |
|  | dibenzo[a,e]fluoranthene | **12.771** | **7.28** | 5385-75-1 |
|  | dibenzo[a,e]pyrene | **13.200** | 7.71 | 192-65-4 |
|  | dibenzo[a,h]anthracene | **11.779** | 6.75 | 53-70-3 |
|  | dibenzo[a,i]pyrene | **12.770** | **7.28** | 189-55-9 |
|  | dibenzo[a,l]pyrene | **13.200** | 7.71 | 191-30-0 |
|  | dibenzothiophene | **7.240** | 4.38 | 132-65-0 |
|  | fluoranthene | **8.601** | 5.16 | 206-44-0 |
|  | fluorene | **6.585** | 4.18 | 86-73-7 |
|  | indeno[1,2,3-cd]pyrene | **11.547** | **6.70** | 193-39-5 |
|  | naphthalene | **5.045** | 3.30 | 91-20-3 |
|  | phenanthrene | **7.222** | 4.46 | 85-01-8 |
|  | pyrene | **8.193** | 4.88 | 129-00-0 |
|  | retene | **8.697** | **6.35** | 483-65-8 |
|  | triphenylene | **10.691** | 5.49 | 217-59-4 |
| Flame | PBDE 100 | **11.977** | **7.66** | 189084-64-8 |
| Retardants | PBDE 153 | 12.150 | **8.55** | 68631-49-2 |
|  | PBDE 154 | **13.265** | **8.55** | 207122-15-4 |
|  | PBDE 28+33 | 9.500/**9.9396** | **5.88/5.88** | 41318-75-6 / 49690-94-0 |
|  | PBDE 47 | 10.530 | **6.77** | 5436-43-1 |
|  | PBDE 99 | 11.310 | 6.84 | 60348-60-9 |
|  | TPP | **8.459** | 4.59 | 115-86-6 |
| Pest. | 4,4'-DDD | 10.100 | 6.02 | 72-54-8 |
|  | 4,4'-DDE | 9.680 | 6.51 | 72-55-9 |
|  | 4,4'-DDT | 9.820 | 6.91 | 50-29-3 |
|  | Alachlor | **9.988** | 3.52 | 15972-60-8 |
|  | Aldrin | 8.080 | 6.50 | 309-00-2 |
|  | alpha-BHC | 8.840 | 3.72 | 319-84-6 |
|  | alpha-Chlordane | 8.982 | 6.16 | 5103-71-9 |
|  | beta-BHC | 8.840 | 3.72 | 319-85-7 |
|  | Bifenthrin | **10.388** | *a* | 82657-04-3 |
|  | Chlorobenzilate | **10.269** | 4.74 | 510-15-6 |
|  | Chloroneb | **6.811** | *a* | 2675-77-6 |
|  | Chloropropylate | **10.894** | *a* | 5836-10-2 |
|  | Chlorothalonil | **7.137** | 3.05 | 1897-45-6 |
|  | Chlorpyrifos | **8.882** | 4.96 | 2921-88-2 |
|  | cis-Permethrin | **10.617** | *a* | 61949-76-6 |
|  | Dacthal | **8.330** | 4.28 | 1861-32-1 |
|  | delta-BHC | 8.840 | 3.72 | 319-86-8 |
|  | Diallate I | **8.299** | 4.49 | 2303-16-4 |
|  | Diazinon | **9.310** | 3.81 | 333-41-5 |
|  | Dieldrin | 8.130 | 5.40 | 60-57-1 |
|  | Dimethoate | **9.147** | 0.78 | 60-51-5 |
|  | Endosulfan I | 8.640 | 3.83 | 959-98-8 |
|  | Endosulfan II | 8.640 | 3.83 | 33213-65-9 |
|  | Endosulfan sulfate | **8.537** | 3.66 | 1031-07-8 |
|  | Endrin | 8.130 | 5.40 | 72-20-8 |
|  | Endrin aldehyde | **8.567** | *a* | 7421-93-4 |
|  | Endrin ketone | **11.073** | *a* | 53494-70-5 |
|  | Esfenvalerate | **10.991** | 6.22 | 66230-04-4 |
|  | Etridiazole | **8.314** | 3.37 | 2593-15-9 |
|  | gamma-Chlordane | **9.542** | **7.00** | 5103-74-2 |
|  | Heptachlor | 7.640 | 6.10 | 76-44-8 |
|  | Heptachlor epoxide | **8.046** | 4.98 | 1024-57-3 |
|  | Hexachlorobenzene | 7.380 | 5.73 | 118-74-1 |
|  | Lindane | 8.840 | 3.72 | 58-89-9 |
|  | Metolachlor | **9.564** | 3.13 | 51218-45-2 |
|  | Mirex | **8.369** | 6.89 | 2385-85-5 |
|  | o,p'-Dicofol | **13.451** | 5.81 | 10606-46-9 |
|  | p,p'-Dicofol | **10.025** | 5.02 | 115-32-2 |
|  | Pendimethalin | **9.636** | 5.18 | 40487-42-1 |
|  | Pentachloronitrobenzene | **7.383** | 4.64 | 82-68-8 |
|  | Perthane | **8.818** | **6.66** | 72-56-0 |
|  | Propachlor | **7.607** | 2.18 | 1918-16-7 |
|  | trans-Nonachlor | 9.660 | 6.35 | 39765-80-5 |
|  | trans-Permethrin | **12.359** | **7.43** | 61949-77-7 |
|  | Trifluralin | **7.716** | 5.34 | 1582-09-8 |
|  | Triclosan | **11.450** | 4.76 | 3380-34-5 |
| PCBs | PCB 105 | 10.000 | 6.79 | 32598-14-4 |
|  | PCB 114 | **9.403** | **6.98** | 74472-37-0 |
|  | PCB 118 | 9.820 | 7.12 | 31508-00-6 |
|  | PCB 123 | **9.403** | **6.98** | 65510-44-3 |
|  | PCB 126 | **10.350** | 6.98 | 57465-28-8 |
|  | PCB 156 | 9.833 | **7.60** | 38380-08-4 |
|  | PCB 157 | **10.173** | **7.62** | 69782-90-7 |
|  | PCB 167 | 10.053 | **7.50** | 52663-72-6 |
|  | PCB 169 | 9.963 | **7.41** | 32774-16-6 |
|  | PCB 170 | **11.704** | **8.27** | 35065-30-6 |
|  | PCB 189 | **10.953** | **8.27** | 39635-31-9 |
|  | PCB 77 | 9.700 | 6.63 | 32598-13-3 |
|  | PCB 81 | **8.632** | **6.34** | 70362-50-4 |
| VOCs | 1,2,3-Trichlorobenzene | 5.190 | **3.93** | 87-61-6 |
|  | 1,2,3-Trimethylbenzene | **4.409** | **3.63** | 526-73-8 |
|  | 1,2,4-Trichlorobenzene | 4.950 | **3.93** | 120-82-1 |
|  | 1,2,4-Trimethylbenzene | **4.339** | **3.63** | 95-63-6 |
|  | 1,3,5-Trimethylbenzene | **3.865** | **3.63** | 108-67-8 |
|  | 1,3-Dichlorobenzene | 4.120 | **3.28** | 541-73-1 |
|  | 2-Chlorotoluene (1-chloro-2-methylbenzene) | **4.256** | **3.18** | 95-49-8 |
|  | 4-Chlorotoluene (1-chloro-4-methylbenzene) | **4.077** | **3.18** | 106-43-4 |
|  | Benzene | 2.780 | 2.13 | 71-43-2 |
|  | Bromobenzene | **3.986** | **2.88** | 108-86-1 |
|  | Chlorobenzene | 3.310 | **2.64** | 108-90-7 |
|  | Cumene (1-methylethyl-benzene) | 3.980 | **3.45** | 98-82-8 |
|  | Ethylbenzene | 3.740 | **3.03** | 100-41-4 |
|  | n-Butylbenzene | **4.567** | **4.01** | 104-51-8 |
|  | n-Octane | 3.350 | **4.27** | 111-65-9 |
|  | n-Decane | **2.687** | **5.25** | 124-18-5 |
|  | n-Dodecane | **3.573** | **6.23** | 112-40-3 |
|  | n-Nonane | **3.507** | **4.76** | 111-84-2 |
|  | n-Pentadecane | **4.998** | **7.71** | 629-62-9 |
|  | n-Propylbenzene | 4.090 | **3.52** | 103-65-1 |
|  | n-Tetradecane | **4.625** | **7.22** | 629-59-4 |
|  | n-Undecane | **4.625** | **5.74** | 1120-21-4 |
|  | o-Dichlorobenzene (1,2-dichlorobenzene) | 4.360 | **3.28** | 95-50-1 |
|  | o-Xylene | 3.910 | **3.09** | 95-47-6 |
|  | p-Dichlorobenzene (1,4-dichlorobenzene) | 4.460 | **3.28** | 106-46-7 |
|  | p-Isopropyltoluene | **4.395** | **4.00** | 99-87-6 |
|  | sec-Butylbenzene (1-methylpropyl benzene) | **4.713** | **3.94** | 135-98-8 |
|  | Styrene | **3.899** | **2.89** | 100-42-5 |
|  | tert-Butylbenzene | **4.378** | **3.90** | 98-06-6 |
|  | Toluene | 3.310 | **2.54** | 108-88-3 |
|  | Xylenes (m and p) | 3.780/3.790 | **3.09/3.09** | 108-38-3 / 06-42-3 |

*^a^* Data not available

**Table S2.** Analytical instrument parameters

|  | ***PAHs*** | ***Flame Retardants*** | ***Pesticides*** | ***PCBs*** | ***VOCs*** |
| --- | --- | --- | --- | --- | --- |
| ***Extraction Surrogate Standards*** | naphthalene-d8, acenaphthylene-d8,  phenanthrene-d10,  fluoranthene-d10,  chrysene-d12,  benzo[a]pyrene-d12,  benzo[ghi]perylene-d12 | fluoro-PBDE 118  2-bromobiphenyl | tetrachloro-meta-xylene,  PCB 100  PCB 209 | phenanthrene-d10,  fluoranthene-d10,  chrysene-d12 | n/a |
| ***Internal Standard*** | perylene-d12 | fluoro-BDE 126 | 4,4’-dibromooctafluoro-biphenyl | perylene-d12 | benzene-d6, toluene-d8, 1,4-dichlorobutane-d8, 1,4-dichlorobenzene-d4 |
| ***Gas Chroma-tograph*** | Agilent 7809 | Agilent 7890A | Agilent 6890N | Agilent 7890A | Markes M-CTE250 & Unity Series 2 Thermal Desorber with Agilent 6890N GC |
| ***Detector(s)*** | 7000C MS/MS | Agilent 5975C | 2x micro-electron  capture detectors | Agilent 5975C | Agilent 5975B |
| ***Column(s)*** | PAH select (Agilent) | DB5-MS (Agilent) | DB-XLB and DB-17MS (both Agilent) | DB5-MS (Agilent) | DB-624, [*2 and 7 day 30°C data sets* DB5-MS] (both Agilent) |
| ***No. of calibration points*** | 5-9 | 5-6 | 5 | 6 | 5-8 |
| ***Temperature program*** | hold 60°C for 1 min,  ramp 40°C/min to 180°C,  3°C/min to 230°C,  1.5°C/min to 280°C,  hold for 10 min,  ramp 6°C/min to 298°C  ramp 16°C/min to 350°C,  hold at 350°C for 4 min  *Total time = 47.25 min* | hold 90°C for 1.25 min,  ramp 10°C/min to 240°C,  ramp 20°C/min to 310°C,  10 min hold  *Total time = 29.75 min* | hold 110°C, 0.5 min.  ramp 25°C/min to 150°C,  ramp 6°C/min to 229°C, ramp 20°C/min to 320°C,  2.5 min hold  *Total time = 22.32* | hold 60°C for 1 min,  ramp 10°C/min to 180°C,  ramp 6°C/min to 310°C  5 min hold  *Total time = 39.67 min* | hold 35°C for 1 min, ramp 20°C/min to 250°C, 7 min hold  [*2 and 7 day 30°C data sets:* hold 35°C for 4 min, ramp 8°C/min to 100, then ramp 16°C/min to 340°C, 4 min hold]  *Total time = 31.13 min* |
| ***Reference*** | Anderson et al. (1) | Kile et al. (2) | Donald et al. (3) | new | new |

**Table S3**: Analytical parameters for micro-chamber thermal extraction M-CTE250 and Unity 2 (Markes, International, Inc.)

^a^ *2 and 7 day 30°C data sets:* Ambient (~20°C) to 50°C for 30 min, 50°C to 250°C for 90 min

^b^ *2 and 7 day 30°C data sets*: 320°C, 12 min hold

^c^ *2 and 7 day 30°C data sets*: Flow Path Temp: 210°C

**Wristband Infusion Additional Details:**

*Infusion and extraction details:* Semi-volatile organic chemicals (SVOCs) are defined as chemicals with boiling points from 250 to 450°C, and volatile organic chemicals (VOCs) as having boiling points less than 250°C (484°F) (4). A mixture of SVOCs in organic solvent were applied to the wristbands, the solvent was allowed to dry for 5 min. and the wristbands were placed in air-tight PTFE bags until analysis. The VOC infusions were performed by placing wristbands in a jar, VOC standards were placed *adjacent* to the wristbands and the jar was immediately sealed for a minimum of 120 minutes. Following infusion, samplers were immediately moved to dark, temperature-controlled environments at temperature conditions discussed above. Ambient light was minimized during laboratory preparation steps. The PTFE bags used in this study attenuate UVA and UVB transmittance by 49% (5).

*Surrogates.* Tetrachloro-meta-xylene, PCB-100 and PCB-209 (Accustandard, USA) were used as extraction surrogate standards for pesticides, and naphthalene-d8, acenaphthalene-d8, phenanthrene-d10, fluoranthene-d10, chrysene-d12, benzo[a]pyrene-d12 and benzo[ghi]perylene-d12 were used for the PAH method (CDN Isotopes, Canada). Phenanthrene-d10, fluoranthene-d10, chrysene-d12 were used as surrogates for PCBs. Internal standards 4,4’-dibromooctafluorobiphenyl (Supelco Analytical, USA) perylene-d12 (Chemservice, USA) and FBDE-126 and FBDE-118 (Accustandard, USA) used for pesticides, PAHs and PCBs, and flame retardants respectively, were added immediately before instrumental analysis to correct for instrument variation (Table S2).

**Paired wristband and active air monitoring backpack PUF Study additional details:**

Informed consent was obtained in agreement with the Columbia University Institutional Review Board (IRB), the IRB of record. Sampling dates are listed in the Table S4.

*Wristband Analysis.* The wristband samples were quantitatively analyzed for 62 PAHs with an Agilent 7890A gas chromatograph interfaced with a modified Agilent 7000 GC-MS/MS, as described elsewhere (1). Instrumental limits of detection are reported in Anderson et al., and range from 0.24 to 6.44 ng extract^-1^. One wristband extract was comprised and not included in analysis, leaving 22 sets of samples from participants.

*Wristband QC.* QC samples represent 56% of the wristband samples analyzed. QC for wristbands included trip blanks, construction blanks, extraction blanks, non-deployed wristbands, continuing calibration verifications (CCV), and extraction surrogates. All blank QC’s were below detection limit for 56 of the 62 PAHs, and any detects were averaged and subtracted from sample concentrations. Laboratory surrogate recoveries ranged from 56% to 93%, with an average recovery of 78%. Instrument concentrations were all surrogate-corrected, and all instrument blanks were below LOD for all PAHs. Before instrumental analyses proceeded, all CCVs were verified at ±20% of the true value for >80% of the PAHs.

*Air Monitoring Backpack PUF Deployment.* The sampling set-up was placed in a backpack, and the sampling head was attached to the shoulder strap of the backpack in order to be close to the individual’s breathing zone while worn. Before each sampling period, the field staff calibrated and leak-tested each air monitoring backpack. Participants were instructed not to turn-off the air monitoring backpack and to phone staff immediately in the event of backpack equipment failure (e.g. battery drains or pump fails). Staff instructed the participants to wear the backpack active sampler and a wristband for all waking hours, and could hang the sampler bag on a nearby chair while sleeping. The pumps on the air monitoring backpacks operated continuously for the entire sampling period at approximately 4 L minute^-1^. The PUF was pre-cleaned, extracted and analyzed at Southwest Research Institute (SWRI, San Antonio, TX) for 20 PAHs.

Unlike the described PUF backpacks, the wristbands are passive samplers from which we cannot define a sampling rate at this time (i.e. daily sampled air volume).

*PUF Extraction and Analysis.* The samples were initially stored at SWRI at -4°C until extraction. Each sample was soxhlet-extracted with 6% diethyl ether in hexane for at least 16 hours, and concentrated to a final extract volume of 1 mL. The samples were analyzed for 20 PAHs with an Agilent 6890 GC and 5973 Mass Selective Detector. The instrumental LOD for each target PAH is 1.0 ng extract^-1^.*Air Monitoring Backpack QC*. After take-down, each backpack underwent a quality control sampling check, factoring in compliance metrics such as duration of sampling time and air flow. A trip and field blank were sent from the CCCEH to SWRI for analysis in combination with the deployed PUF samples. At SWRI, each sample extract was spiked with an extraction surrogate solution (1-methylnaphthalene-d_10_ and p-terphenyl-d_14_). Recoveries of 1-methylnaphthalene-d_10_ ranged from 87% to 111%, and recoveries of p-terphenyl-d_14_ ranged from 112% to 133%. Two matrix blanks and two matrix spikes of all targeted individual PAHs were also prepared and analyzed. Deuterated PAHs were used as internal standards during instrument analysis. Naphthalene, 2-methylnaphthalene, 1-methylnapthalene, and phenanthrene were present in concentrations above LOD in the PUF matrix blanks. The PAH concentrations in the matrix blanks were subtracted from the measured amount of that PAH in the samples.

*Calculating K_sa_*. First, for each backpack sampler, the volume of air sampled (L) was determined by multiplying the mean air flow rate (L min^-1^) by the time run (min). Air volume sampled by PUF was specific for each study participant, and ranged from 3.99 to 4.05 L min^-1^. K_sa_ values for each PAH were then calculated using

$K_{\mathrm{sa}}\left( L kg^{-1} \right)= \frac{{PAH mass in wristband (ng)}/{mean wristband mass (kg)}}{{PAH mass in PUF (ng)}/{air volume sampled by PUF (L)}}$ $K_{sa}(L {kg}^{-1})=\frac{\frac{PAH mass in wristband (ng)}{mean wrsitband mass (kg)}}{\frac{PAH mass in PUF (ng)}{air volume sampled by PUF (L)}}$ Eq. S1

For the twelve PAHs included in the K_sa_ calculation, the average sum PAH concentration in the wristbands was 1085 ng/wristband (ranging from 15 ng/wristband for acenaphthylene to 298 ng/wristband for phenanthrene). The average sum PAH concentration in the PUFs was 1681 ng/PUF (ranging from 33 ng/PUF for pyrene to 544 ng/PUF for phenanthrene). For all wristbands used in this study, the average wristband mass was 5.7g. **Table S4.** Dates of paired wristband and active air monitoring backpack PUF study.

| Participant | Date at End of 48 Hour  Study Period |
| --- | --- |
| **1** | 11/13/2013 |
| **2** | 12/03/2013 |
| **3** | 12/03/2013 |
| **4** | 12/11/2013 |
| **5** | 01/06/2014 |
| **6** | 01/15/2014 |
| **7** | 03/10/2014 |
| **8** | 03/10/2014 |
| **9** | 03/18/2014 |
| **10** | 07/22/2014 |
| **11** | 08/12/2014 |
| **12** | 09/17/2014 |
| **13** | 09/23/2014 |
| **14** | 10/08/2014 |
| **15** | 10/14/2014 |
| **16** | 11/05/2014 |
| **17** | 11/24/2014 |
| **18** | 12/17/2014 |
| **19** | 01/13/2015 |
| **20** | 03/31/2015 |
| **21** | 07/21/2015 |
| **22** | 08/05/2015 |

**
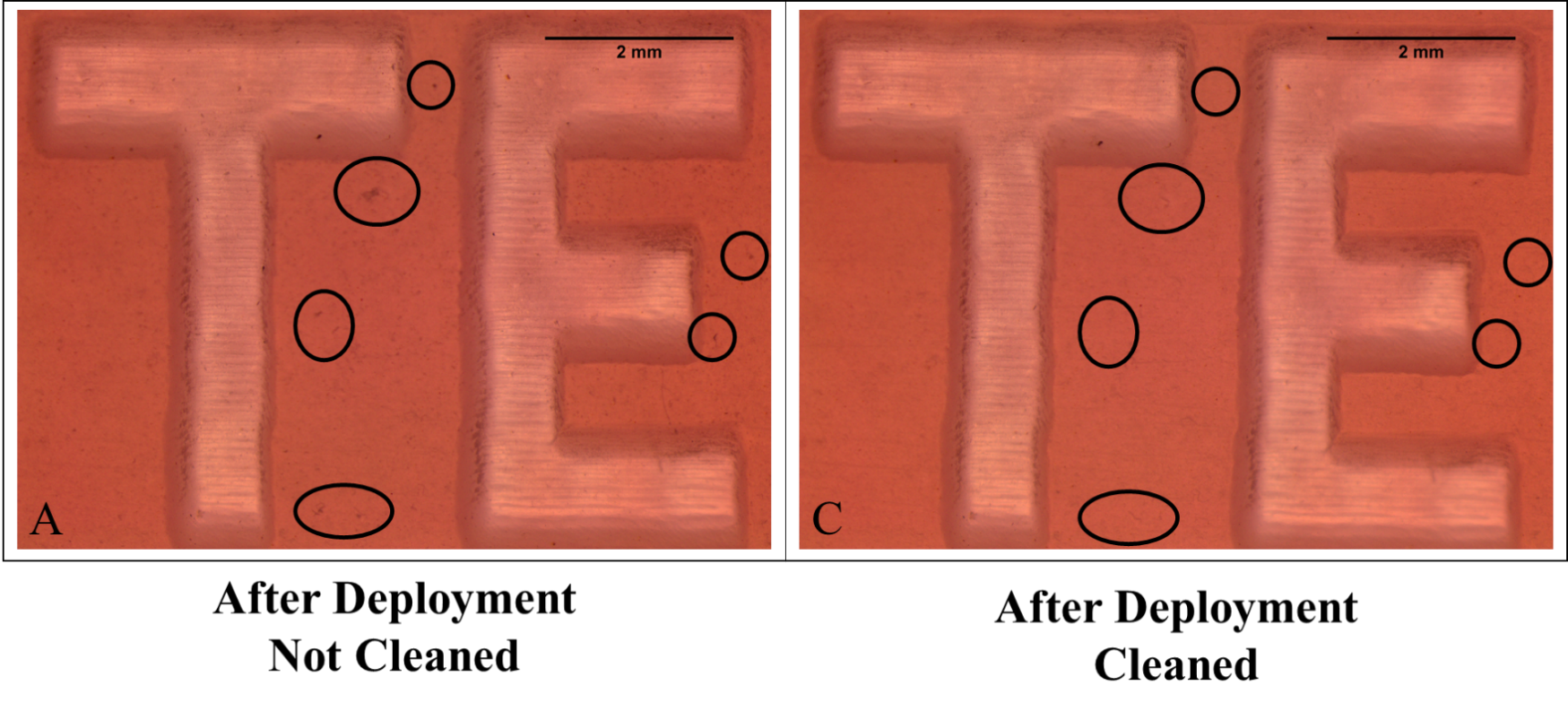

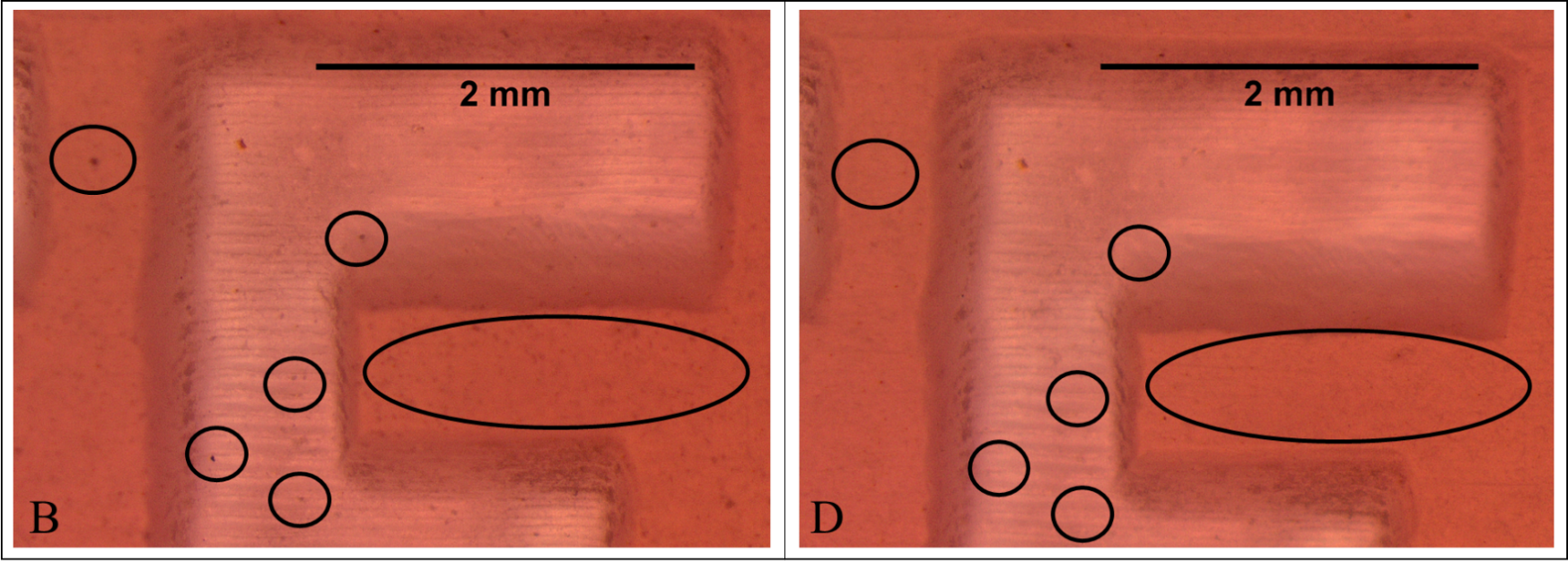
**

**Figure S1.** Microscopic images of a wristband after being worn for 48 hours. SXA-B includes images of the wristband after deployment, but before the cleaning process. SXC-D includes images of the same wristband after the cleaning process. Black ovals highlight key differences in particulates on the wristband surface before and after cleaning. Particulates removed by cleaning account for an area of 1.5 mm^2^, which is 3% of the wristband surface pictured in S1C-D, which indicates that the percentage of particulates on both cleaned and uncleaned wristbands is quite low. Pictures were captured on an Olympus Microscope SZX10-ILLK (Olympus Corporation, Tokyo, Japan) using Olympus cellSens software (version 1.11) and analyzed using ImageJ software (version 1.49; National Institutes of Health, Bethesda, Maryland). Particulates on the wristband surface were also quantitated using ImageJ. For all pictures, contrast was increased by 7%, brightness was increased by 14%, and a gamma correction of 1.15 was applied.

**Conditioned wristbands integrity details:**

After undergoing the heat conditioning process, the wristbands decrease in mass from heat vacuum conditioning 4.93 to 4.74 g, (RSD <1%) Figure S2A. The difference between wristband locations within the oven is less than 1% (Figures S2B) and had no effect on the chromatography. The mass reduction when conditioning was optimized was a nominally a 4% reduction in weight Figure S2C. This criterion was found to be a good balance of maintaining structural integrity of the wristband and removal of sufficient oligomers and other chemical contaminants. Unconditioned wristbands have significant chromatographic interference, after conditioning, chromatography is significantly improved; see Figure 1 for chromatograms. The TIC does not have any unresolved complex mixture and the TIC has four or fewer discrete peaks 15 times greater than our internal standard of perylene-d12 at 500 ng for the clean wristband.

Heat conditioning does not adversely affect the strength and durability of wristband PSDs. Strength of the wristband was assessed by employing a Rapala® 50-pound maximum load scale with a retaining point and applying continuous force until wristband failure. Prior to conditioning wristbands broke at 16.8 ± 0.8 kg of applied pull. Conditioned wristband broke at 9.6 ± 0.1 kg of applied pull (See Table S5A and B for summary statistics and Figure S3A for apparatus). Elasticity was assessed by repeatedly stretching the wristband out 12.7 cm for 300 repetitions, summary statistics provided in Table S5 C (see Figure S3B for apparatus). Heat conditioned wristbands increased in diameter by 1.69 ± 0.14 cm after 300 repetitions but did not break; no further increase in diameter occurred with 200 additional repetitions.

A.
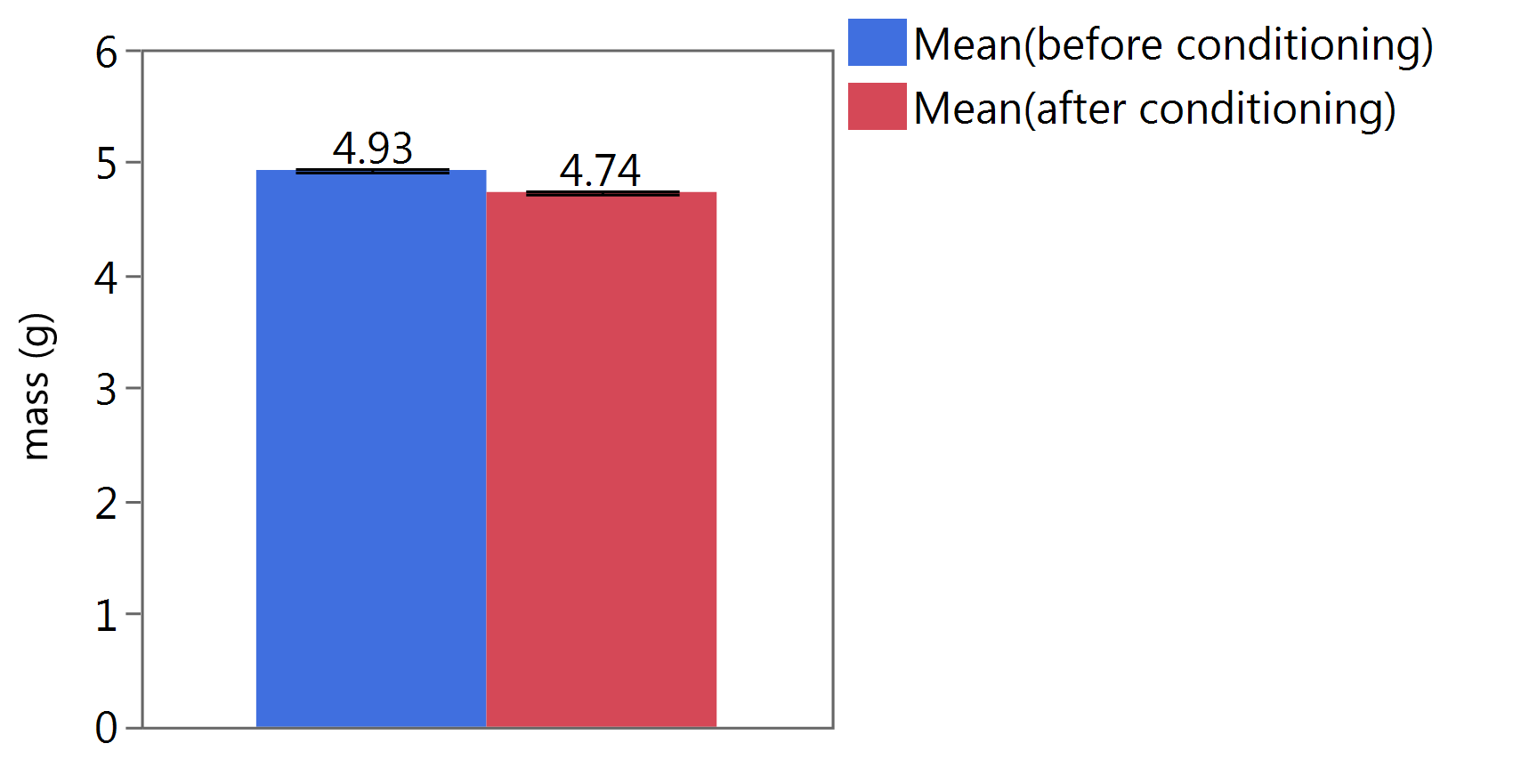


B.
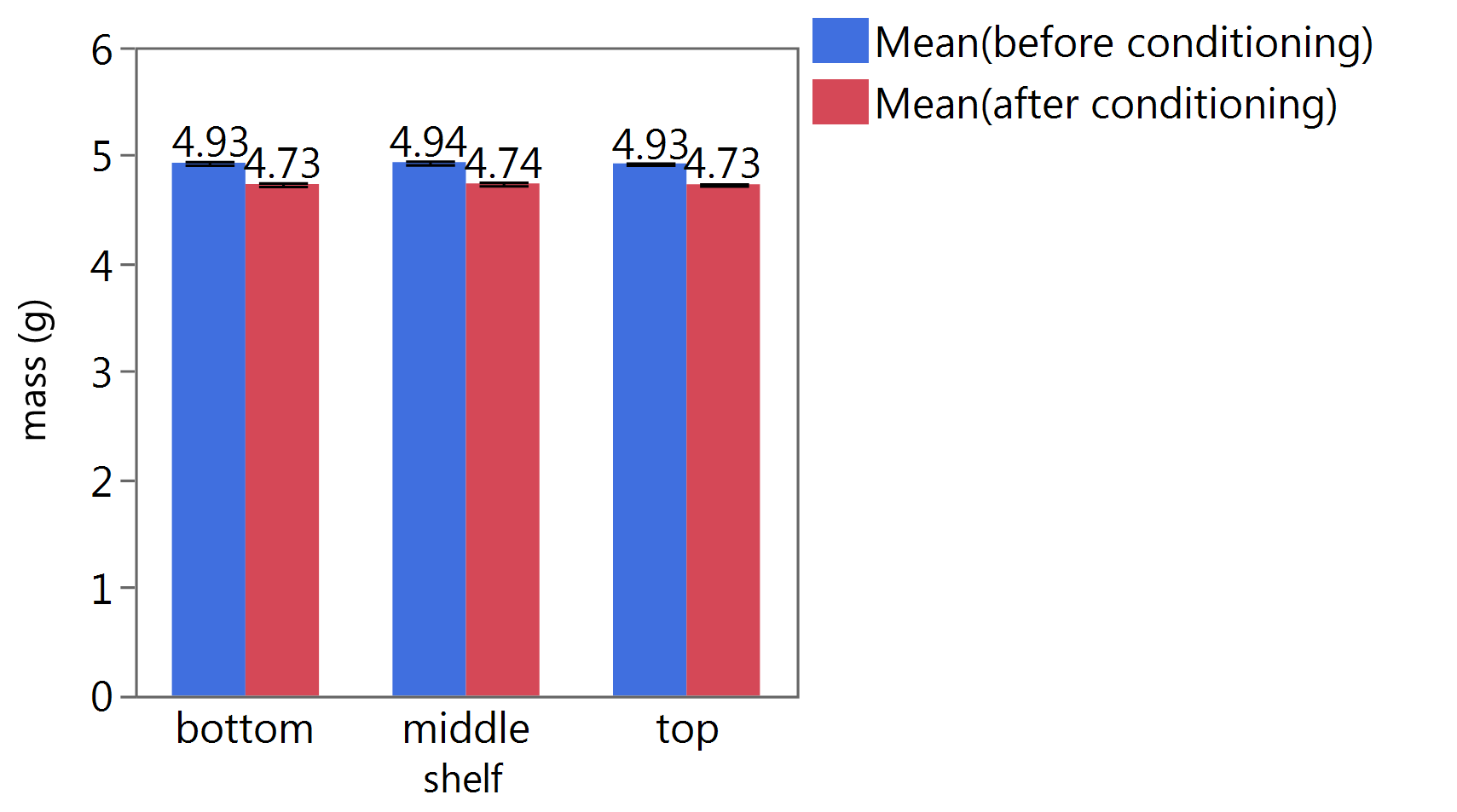


C.
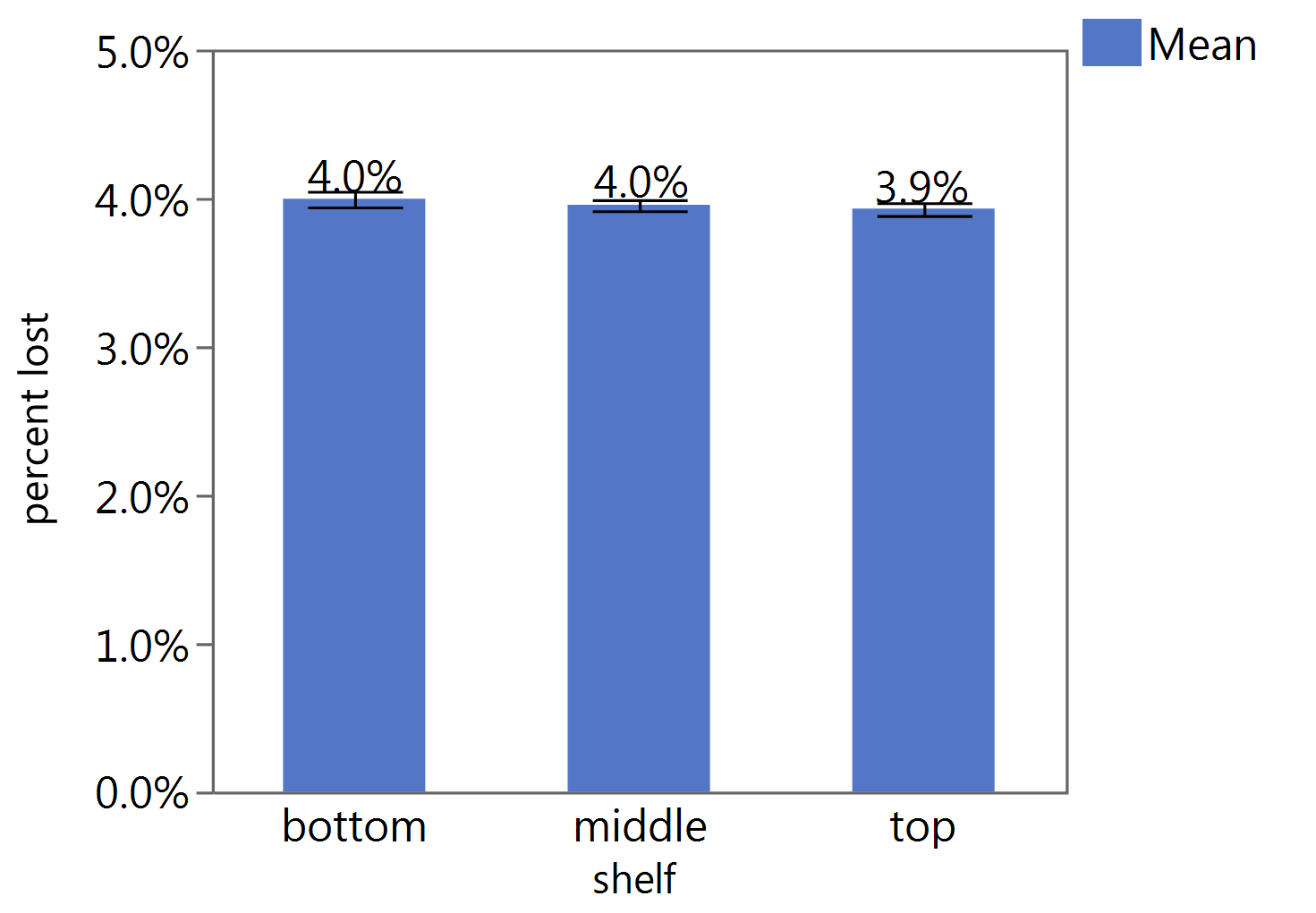


**Figure S2**: **A**) Mean mass before and after conditioning for n=60 wristbands, **B**) mean mass before and after condition for each shelf in the vacuum oven n=20, 20 and 20 bottom, middle and top shelf respectively; **C**) average percentage loss for wristbands by shelf. All errors bars (a, b, and c) depict one standard deviation.

**
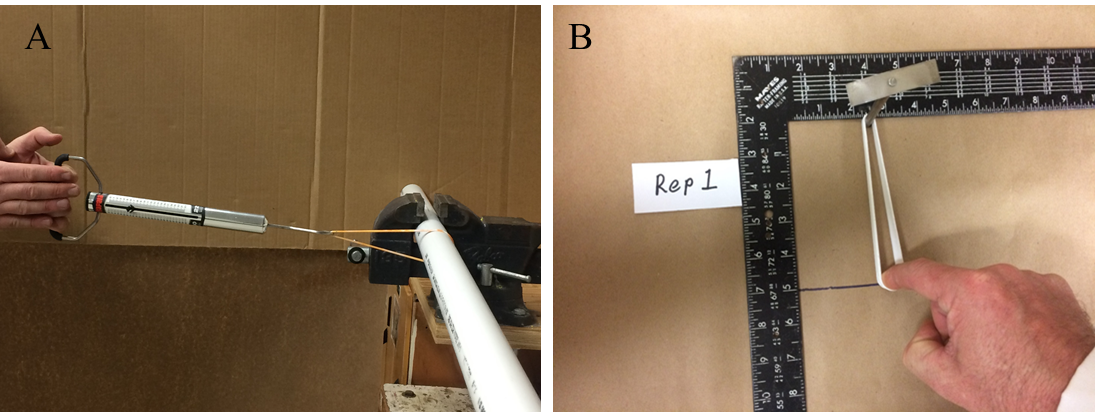
**

**Figure S3 A)** Rapala® 50-pound maximum load scale with a retaining point. **B)** Elasticity was assessed by repeatedly stretching the PSDs a set distance and a set amount of repetitions.

**Table S5:** **A)** measurement of heat conditioned wristband strength **B)** measurement of non-heat conditioned wristband strength **C)** measurement of wristband elasticity.

| **A. Heat conditioned** | **Break Point (kg)** | **Average** | **SD** |
| --- | --- | --- | --- |
| **replicate 1** | 9.5 | 9.6 | 0.1 |
| **replicate 2** | 9.7 |  |  |
| **replicate 3** | 9.6 |  |  |

| **B. Non-heat conditioned** | **Break Point (kg)** | **Average** | **SD** |
| --- | --- | --- | --- |
| **replicate 1** | 17.7 | 16.8 | 0.8 |
| **replicate 2** | 16.1 |  |  |
| **replicate 3** | 16.6 |  |  |

| 1. **Elasticity** | | | | | | | |
| --- | --- | --- | --- | --- | --- | --- | --- |
| **Heat Conditioned** | **Start Diameter (cm)** | **100 Pulls Diameter (cm)** | **200 Pulls Diameter (cm)** | **300 Pulls Diameter (cm)** | **500 Pulls Diameter (cm)** | **Average** | **SD** |
| **replicate 1** | 6.35 | 7.87 | 8.13 | 8.13 | 8.13 | 8.04 | 0.15 |
| **replicate 2** | 6.35 | 7.87 | 7.87 | 7.87 | 7.87 |  |  |
| **replicate 3** | 6.35 | 7.87 | 8.13 | 8.13 | 8.13 |  |  |

**Figure S4.** Individualized linear regression plots of log K_oa_ and estimated log K_sa_. Data from each of 22 participants are shown.


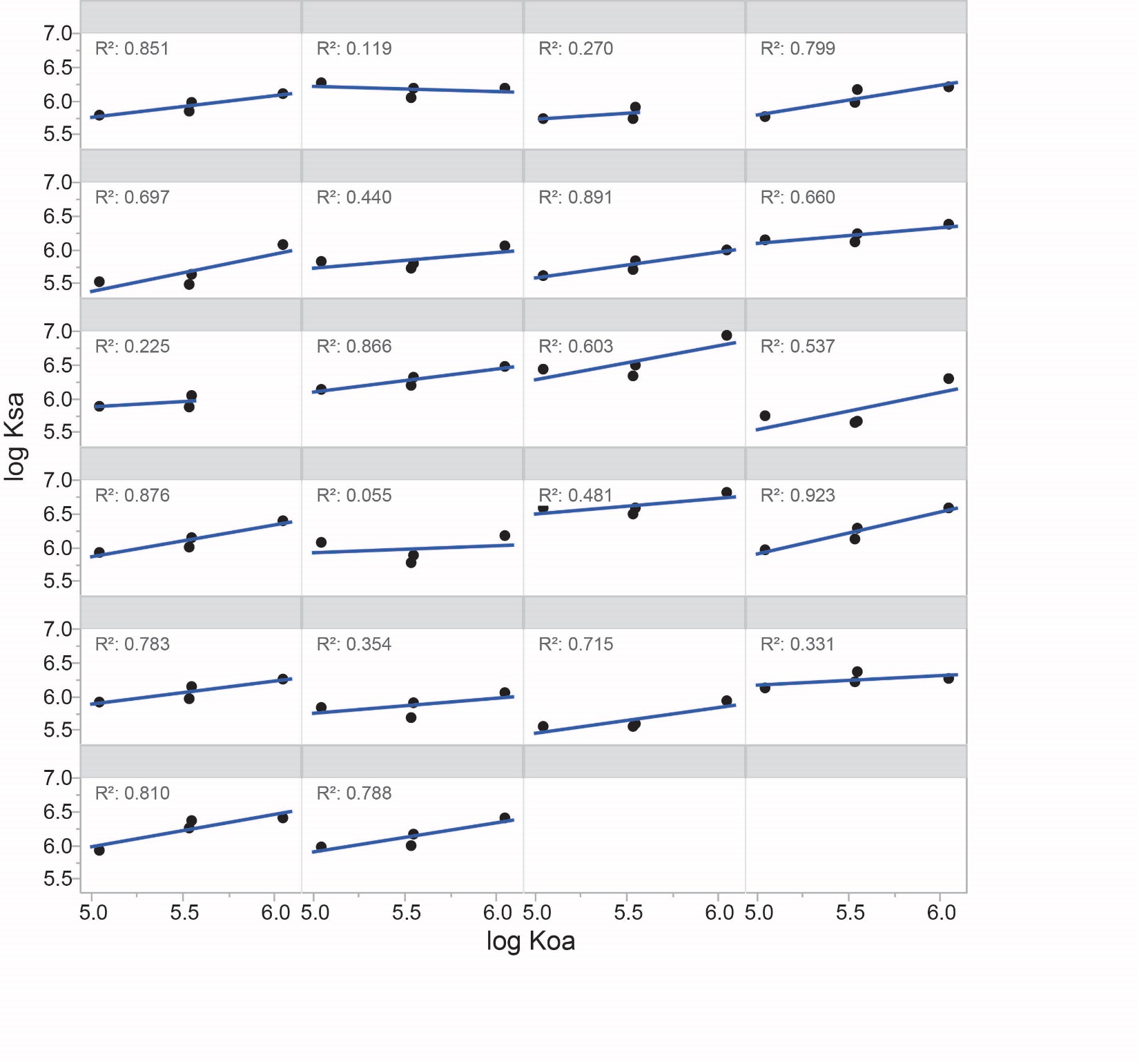


**Table S6.** Average recovery for each time and temperature scenario (n=4 unless otherwise designated). Values less than 70% are highlighted in red, and values greater than 130% are in blue. Bold indicates significantly different than t=0 control, and “--" indicates not tested.

|  |  | **2-4 days** | | | **1 week** | | | **2 weeks** | **1 month** | | | **3 months** | **6 months** |
| --- | --- | --- | --- | --- | --- | --- | --- | --- | --- | --- | --- | --- | --- |
|  |  | **-20°C** | **4°C** | **30°C** | **-20°C** | **4°C** | **30°C** | **4°C** | **-20°C** | **4°C** | **30°C** | **-20°C** | **-20°C** |
| **PAHs** | 1,2-dimethylnaphthalene | **142%** | **138% ^a^** | **134%** | 86% | 81% | 84% | -- | 102% | 99% | 115% | 111% | 95% |
|  | 1,4-dimethylnaphthalene | **138%** | **139% ^a^** | **137%** | 84% | 78% | 84% | -- | 101% | 98% | 115% | 102% | 99% |
|  | 1,5-dimethylnaphthalene | **140%** | **135% ^a^** | **135%** | 90% | 80% | 87% | -- | 107% | 101% | 127% | 118% | 98% |
|  | 1,6-dimethylnaphthalene | **146%** | **136% ^a^** | **137%** | 87% | 83% | 88% | -- | 102% | 103% | 120% | 118% | 105% |
|  | 1,8-dimethylnaphthalene | **144%** | **134% ^a^** | **136%** | 86% | 81% | 88% | -- | 108% | 103% | 124% | 111% | 98% |
|  | 1-methylnaphthalene | **141%** | **133% ^a^** | **136%** | 86% | 80% | 87% | -- | 107% | 102% | 122% | 113% | 95% |
|  | 1-methylphenanthrene | 93% | 95% **^a^** | 98% | 91% | 89% | 90% | -- | 113% | 108% | 114% | 97% | 112% |
|  | 1-methylpyrene | 99% | 103% **^a^** | 101% | 92% | 99% | 95% | -- | **124%** | **121%** | **128%** | 106% | 104% |
|  | 2,3-dimethylanthracene | **78%** | 82% **^a^** | **78%** | **83%** | **77%** | **78%** | -- | 97% | 101% | 105% | 102% | 89% |
|  | 2,6-diethylnaphthalene | **144%** | 135% **^a^** | **135%** | 86% | 79% | 84% | -- | 102% | 99% | 113% | 117% | 110% |
|  | 2,6-dimethylnaphthalene | 137% | 127% **^a^** | 128% | 85% | 77% | 84% | -- | 97% | 96% | 107% | 117% | 99% |
|  | 2-ethylnaphthalene | **144%** | 129% **^a^** | 133% | 86% | 77% | 79% | -- | 99% | 96% | 118% | 110% | 101% |
|  | 2-methylanthracene | 93% | 99% **^a^** | 102% | 99% | 90% | 94% | -- | **115%** | 111% | **116%** | 93% | **118%** |
|  | 2-methylnaphthalene | **137%** | 127% **^a^** | **132%** | 84% | 76% | 80% | -- | 100% | 96% | 112% | 119% | 97% |
|  | 2-methylphenanthrene | 101% | 95% **^a^** | 101% | 96% | 94% | 94% | -- | **119%** | **114%** | **120%** | 102% | **122%** |
|  | 3,6-dimethylphenanthrene | 92% | 95% **^a^** | 98% | 93% | 94% | 97% | -- | **116%** | **124%** | **130%** | **126%** | 94% |
|  | 6-methylchrysene | 98% | 96% **^a^** | 101% | 91% | 93% | 93% | -- | **116%** | **118%** | **122%** | **116%** | 101% |
|  | 7,12-dimethylbenz[a]anthracene | 98% | 99% **^a^** | 94% | 96% | 99% | 91% | -- | **131%** | **124%** | **120%** | **85%** | 92% |
|  | 9,10-dimethylanthracene | 82% | 91% **^a^** | 81% | 93% | 104% | 101% | -- | **132%** | **148%** | **150%** | 77% | 85% |
|  | 9-methylanthracene | 87% | 88% **^a^** | 89% | 90% | **83%** | **84%** | -- | 105% | 105% | 108% | 90% | 110% |
|  | acenaphthene | 156% | 138% **^a^** | 140% | 97% | 84% | 90% | -- | 120% | 118% | 128% | 88% | 119% |
|  | acenaphthylene | 117% | 120% **^a^** | 121% | 89% | 84% | 92% | -- | 113% | 96% | 109% | 98% | 112% |
|  | anthanthrene | **67%** | **68% ^a^** | **66%** | 79% | **66%** | **72%** | -- | 88% | 95% | 89% | **73%** | **133%** |
|  | Anthracene | 105% | 104% **^a^** | 106% | 98% | 94% | 92% | -- | 119% | 112% | 118% | 106% | **132%** |
|  | benz[a]anthracene | 94% | 93% **^a^** | 90% | **84%** | 87% | **86%** | -- | **115%** | 112% | **115%** | 100% | 107% |
|  | benzo[a]chrysene | 90% | 100% **^a^** | 87% | 86% | 87% | 89% | -- | 116% | 113% | 107% | 100% | 118% |
|  | benzo[a]pyrene | 99% | 94% **^a^** | 93% | **87%** | 96% | 95% | -- | **126%** | **117%** | **125%** | 91% | 102% |
|  | benzo[b]fluoranthene | 95% | 94% **^a^** | 95% | 91% | 91% | 91% | -- | **116%** | **112%** | **115%** | 102% | 105% |
|  | benzo[c]fluorene | 96% | 95% **^a^** | 92% | **88%** | **88%** | **89%** | -- | **115%** | **112%** | **115%** | 99% | 103% |
|  | benzo[e]pyrene | 98% | 97% **^a^** | 98% | **88%** | 93% | 94% | -- | **121%** | **117%** | **123%** | **89%** | 99% |
|  | benzo[ghi]perylene | 91% | 93% **^a^** | 91% | **85%** | 90% | 89% | -- | 106% | 103% | 100% | 89% | **126%** |
|  | benzo[j]fluoranthene | 93% | 94% **^a^** | 89% | 93% | 94% | 96% | -- | **125%** | **122%** | **129%** | **81%** | **85%** |
|  | benzo[k]fluoranthene | 102% | 112% **^a^** | 96% | 94% | 95% | 89% | -- | **124%** | 112% | **132%** | 111% | 120% |
|  | Chrysene | 104% | 97% **^a^** | 100% | 90% | 100% | 96% | -- | **128%** | **124%** | **126%** | 99% | 102% |
|  | Coronene | 87% | 84% **^a^** | 85% | 89% | 93% | 92% | -- | 104% | 104% | 104% | **65%** | **142%** |
|  | cyclopenta[cd]pyrene | 90% | **85% ^a^** | **79%** | **77%** | **76%** | **80%** | -- | 96% | 90% | 91% | **69%** | **88%** |
|  | dibenzo[a,e]fluoranthene | **84%** | 88% **^a^** | **88%** | 94% | 102% | 93% | -- | **121%** | **114%** | **121%** | **73%** | **124%** |
|  | dibenzo[a,e]pyrene | 96% | 93% **^a^** | 88% | 95% | 95% | 103% | -- | **118%** | 115% | 115% | **78%** | 115% |
|  | dibenzo[a,h]anthracene | 90% | 98% **^a^** | 92% | 85% | 88% | 87% | -- | 104% | 109% | 112% | 100% | 110% |
|  | dibenzo[a,i]pyrene | 91% | 95% **^a^** | **80%** | 95% | 96% | 96% | -- | 113% | 108% | **123%** | **75%** | **126%** |
|  | dibenzo[a,l]pyrene | 92% | 105% **^a^** | 93% | 91% | 99% | 96% | -- | **114%** | **124%** | **119%** | 90% | **116%** |
|  | dibenzothiophene | 97% | 101% **^a^** | 104% | 94% | 92% | **90%** | -- | **117%** | **113%** | **115%** | 101% | **116%** |
|  | fluoranthene | 93% | 91% **^a^** | 97% | **86%** | **87%** | 89% | -- | **114%** | 109% | **114%** | 89% | 107% |
|  | Fluorene | **135%** | 122% **^a^** | **127%** | 81% | **73%** | 79% | -- | 93% | 97% | 110% | 107% | 103% |
|  | indeno[1,2,3-cd]pyrene | 102% | 103% **^a^** | 94% | 92% | 96% | 90% | -- | 107% | 103% | 113% | 105% | 105% |
|  | naphthalene | **138%** | 122% **^a^** | **137%** | 82% | 75% | 85% | -- | 104% | 99% | 114% | 109% | 97% |
|  | phenanthrene | 96% | 97% **^a^** | 105% | 98% | 92% | **87%** | -- | **113%** | 112% | **116%** | 107% | **125%** |
|  | Pyrene | 103% | 101% **^a^** | 108% | 103% | 104% | 100% | -- | **129%** | **125%** | **129%** | 85% | 99% |
|  | Retene | 91% | 96% **^a^** | 91% | **84%** | 93% | 93% | -- | **117%** | **114%** | **126%** | 106% | 94% |
|  | triphenylene | 99% | 99% **^a^** | 98% | 90% | 90% | 95% | -- | **118%** | **113%** | **123%** | 94% | 100% |
| **FRs** | PBDE 100 | 108% | 110% | 102% | 104% | 103% | 104% | -- | 92% | 98% | 100% | 108% | 108% |
|  | PBDE 153 | 102% | 108% | 101% | 94% | 98% | 101% | -- | 93% | 93% | 100% | 108% | 101% |
|  | PBDE 154 | 108% | 108% | 104% | 102% | 104% | 108% | -- | 102% | 101% | 101% | 110% | 104% |
|  | PBDE 28+33 | 107% | 107% | 104% | 105% | 103% | 106% | -- | 104% | 105% | 103% | 106% | 104% |
|  | PBDE 47 | 106% | 105% | 99% | 102% | 99% | 102% | -- | 92% | 94% | 100% | 104% | 99% |
|  | PBDE 99 | 108% | 102% | 103% | 102% | 108% | 108% | -- | 99% | 98% | 100% | 103% | **74%** |
|  | TPP | 101% | 97% | 97% | 107% | 101% | 95% | -- | 96% | 94% | 97% | 98% | **78%** |
| **Pest.** | 4,4'-DDD | -- | -- | -- | 101% | 101% | 91% | -- | 103% | 109% | **124%** | 95% | **124%** |
|  | 4,4'-DDE | -- | -- | -- | 101% | 104% | 94% | -- | 111% | 115% | **127%** | 101% | **124%** |
|  | 4,4'-DDT | -- | -- | -- | 83% | 101% | 95% | -- | 96% | 104% | **123%** | 93% | **129%** |
|  | Alachlor | -- | -- | -- | 98% | 100% | 90% | -- | **119%** | 113% | **120%** | 92% | 115% |
|  | Aldrin | -- | -- | -- | 98% | 100% | 101% | -- | **121%** | 116% | **120%** | 86% | 115% |
|  | alpha-BHC | -- | -- | -- | 98% | 99% | 98% | -- | **122%** | **115%** | **121%** | 92% | **118%** |
|  | alpha-Chlordane | -- | -- | -- | 102% | 105% | 94% | -- | 112% | 115% | **127%** | 97% | **124%** |
|  | beta-BHC | -- | -- | -- | 94% | 98% | 99% | -- | **120%** | 114% | **122%** | 91% | **115%** |
|  | Bifenthrin | -- | -- | -- | 101% | 98% | 101% | -- | 107% | 115% | 114% | 91% | 116% |
|  | Chlorobenzilate | -- | -- | -- | 98% | 103% | 96% | -- | 105% | 111% | **125%** | 99% | **122%** |
|  | Chloroneb | -- | -- | -- | 93% | 99% | 95% | -- | 114% | 112% | **124%** | 97% | 113% |
|  | Chloropropylate | -- | -- | -- | 98% | 102% | 95% | -- | 100% | 103% | **124%** | 93% | **121%** |
|  | Chlorothalonil | -- | -- | -- | 91% | 103% | 93% | -- | 106% | 99% | 104% | 100% | **130%** |
|  | Chlorpyrifos | -- | -- | -- | 99% | 101% | 91% | -- | **125%** | 113% | 93% | 92% | **127%** |
|  | Dacthal | -- | -- | -- | 112% | 121% | 100% | -- | 117% | 117% | 134% | 102% | 131% |
|  | delta-BHC | -- | -- | -- | 100% | 109% | 98% | -- | **119%** | 114% | **122%** | 99% | **126%** |
|  | Diallate I | -- | -- | -- | 98% | 106% | 92% | -- | 113% | 103% | 110% | 79% | **129%** |
|  | Diazinon | -- | -- | -- | 98% | 98% | 96% | -- | 113% | 101% | 112% | **62%** | 107% |
|  | Dieldrin | -- | -- | -- | 102% | 105% | 95% | -- | 107% | 111% | **126%** | 97% | **124%** |
|  | Dimethoate | -- | -- | -- | 93% | 87% | 94% | -- | 116% | 103% | 104% | **65%** | 114% |
|  | Endosulfan I | -- | -- | -- | 96% | 94% | 106% | -- | **128%** | 119% | 112% | 86% | 110% |
|  | Endosulfan II | -- | -- | -- | 104% | 104% | 94% | -- | 105% | 109% | **124%** | 100% | **128%** |
|  | Endosulfan sulfate | -- | -- | -- | 88% | 92% | 85% | -- | 88% | 96% | 115% | 82% | 112% |
|  | Endrin | -- | -- | -- | 104% | 99% | 86% | -- | 107% | 104% | **69%** | 100% | **124%** |
|  | Endrin aldehyde | -- | -- | -- | 100% | 96% | 84% | -- | 77% | 67% | 70% | 84% | 140% |
|  | Endrin ketone | -- | -- | -- | 91% | 97% | 89% | -- | 86% | 99% | **135%** | 90% | **125%** |
|  | Esfenvalerate | -- | -- | -- | **73%** | 87% | 95% | -- | 95% | 92% | 90% | 80% | 105% |
|  | Etridiazole | -- | -- | -- | 97% | 104% | 114% | -- | **125%** | **118%** | **125%** | 99% | **119%** |
|  | gamma-Chlordane | -- | -- | -- | 98% | 98% | 100% | -- | **118%** | 115% | **119%** | 89% | 115% |
|  | Heptachlor | -- | -- | -- | 106% | 112% | 103% | -- | **130%** | **123%** | **128%** | 105% | **130%** |
|  | Heptachlor epoxide | -- | -- | -- | 100% | 104% | 95% | -- | 117% | 117% | **127%** | 99% | **124%** |
|  | Hexachlorobenzene | -- | -- | -- | 100% | **118%** | **120%** | -- | **124%** | 115% | **119%** | 95% | **120%** |
|  | Isodrin | -- | -- | -- | 101% | 106% | 94% | -- | 117% | **117%** | **126%** | 91% | **121%** |
|  | Lindane | -- | -- | -- | 98% | 97% | 95% | -- | **120%** | 114% | **121%** | 94% | **120%** |
|  | Metolachlor | -- | -- | -- | 96% | 88% | 84% | -- | **131%** | 125% | 118% | 97% | 115% |
|  | Mirex | -- | -- | -- | 94% | 95% | **81%** | -- | **69%** | 83% | 112% | **76%** | 118% |
|  | o,p'-Dicofol | -- | -- | -- | 92% | 95% | 92% | -- | **122%** | 116% | **118%** | 93% | 113% |
|  | p,p'-Dicofol | -- | -- | -- | 103% | **158%** | **155%** | -- | 121% | 118% | 126% | 102% | 124% |
|  | Pendimethalin | -- | -- | -- | 107% | **122%** | 109% | -- | **120%** | 118% | **127%** | **79%** | **122%** |
|  | Pentachloronitrobenzene | -- | -- | -- | 96% | 107% | 106% | -- | 113% | 111% | **118%** | 86% | **118%** |
|  | Permethrin | -- | -- | -- | 87% | 97% | 95% | -- | **145%** | 115% | 116% | 99% | 109% |
|  | Perthane | -- | -- | -- | 89% | 99% | 83% | -- | 105% | 103% | **123%** | 85% | 109% |
|  | Propachlor | -- | -- | -- | 102% | 108% | 112% | -- | **125%** | 118% | 112% | 106% | **127%** |
|  | Prophos | -- | -- | -- | 97% | 96% | 96% | -- | 125% | 113% | 112% | **154%** | 137% |
|  | trans-Nonachlor | -- | -- | -- | 97% | 100% | 103% | -- | 119% | 115% | 117% | 89% | 118% |
|  | triclosan | 110% | 118% | 108% | 110% | 101% | 102% | -- | 108% | 108% | 94% **^a^** | 91% | -- |
|  | Trifluralin | -- | -- | -- | 98% | 116% | **123%** | -- | 106% |  |  |  | 115% |
| **PCBs** | PCB 105 | 120% | **134%** | **128%** | **133%** | 120% | **128%** | -- | 119% | **123%** | 118% | **127%** | 120% |
|  | PCB 114 | 121% | **134%** | **131%** | **130%** | **124%** | **128%** | -- | **124%** | **123%** | 112% | **124%** | **130%** |
|  | PCB 118 | 122% | **137%** | **126%** | **132%** | 121% | **125%** | -- | 119% | **124%** | 117% | **126%** | **123%** |
|  | PCB 123 | 118% | **135%** | **126%** | **131%** | 122% | **128%** | -- | 119% | **123%** | 114% | 117% | 120% |
|  | PCB 126 | 115% | 126% | 125% | **140%** | 122% | 121% | -- | 117% | 118% | 104% | 116% | **136%** |
|  | PCB 156 | 119% | 130% | 123% | 138% | 121% | 122% | -- | 114% | 118% | 109% | 120% | **168%** |
|  | PCB 157 | 117% | 129% | 122% | **134%** | 118% | 123% | -- | 114% | 115% | 110% | 121% | **132%** |
|  | PCB 167 | 116% | 128% | 125% | **136%** | 122% | 124% | -- | 118% | 119% | 105% | 130% | 116% |
|  | PCB 169 | 116% | 130% | 123% | **138%** | 119% | 124% | -- | 116% | 117% | 109% | 121% | 128% |
|  | PCB 170 | 116% | 128% | 120% | **137%** | 120% | 122% | -- | 117% | 117% | 107% | 123% | 131% |
|  | PCB 189 | 117% | 130% | 121% | **137%** | 121% | 122% | -- | 117% | 119% | 108% | 124% | 127% |
|  | PCB 77 | 118% | **129%** | 125% | **128%** | **123%** | **125%** | -- | **122%** | **124%** | 112% | **130%** | **130%** |
|  | PCB 81 | 118% | **129%** | **125%** | **124%** | **120%** | **122%** | -- | **120%** | **126%** | 111% | **132%** | **134%** |
| **VOCs** | 1,2,3-Trichlorobenzene | -- | 89% **^a^** | 93% | 92% | 99% **^a^** | **69% ^a^** | 93% | 97% | 97% | 94% | 80% | -- |
|  | 1,2,3-Trimethylbenzene | -- | 98% **^a^** | 99% | 101% | 108% **^a^** | 95% ^a^ | 100% | 95% | 103% | 78% | 105% | -- |
|  | 1,2,4-Trichlorobenzene | -- | 88% **^a^** | 97% | 89% | 97% **^a^** | 94% ^a^ | 94% | 96% | 94% | **73%** | 103% | -- |
|  | 1,2,4-Trimethylbenzene | -- | 101% **^a^** | 82% | 88% | 115% **^a^** | **77% ^a^** | 85% | 87% | 87% | **66%** | 90% | -- |
|  | 1,3,5-Trimethylbenzene | -- | 102% **^a^** | 83% | 90% | 115% **^a^** | **78% ^a^** | 87% | 95% | 91% | **75%** | 91% | -- |
|  | 1,3-Dichlorobenzene | -- | 100% **^a^** | 95% | 89% | **124% ^a^** | **90% ^a^** | 90% | 97% | 91% | **56%** | 98% | -- |
|  | 2-Chlorotoluene | -- | 101% **^a^** | 82% | 90% | 112% **^a^** | **77% ^a^** | 88% | 96% | 94% | **63%** | 93% | -- |
|  | 4-Chlorotoluene | -- | 101% **^a^** | 81% | 85% | 112% **^a^** | **78% ^a^** | 86% | 93% | 89% | **47%** | 92% | -- |
|  | Benzene | -- | -- | 106% | -- | -- | **80% ^a^** | -- | -- | -- | **--** | -- | -- |
|  | Bromobenzene | -- | 103% **^a^** | 81% | 83% | 112% **^a^** | **74% ^a^** | 81% | 91% | 90% | **51%** | 97% | -- |
|  | Chlorobenzene | -- | 114% **^a^** | 96% | 101% | 111% **^a^** | **86% ^a^** | 97% | 94% | **118%** | **31%** | 100% | -- |
|  | Cumene | -- | 104% **^a^** | 99% | 100% | 105% **^a^** | **94% ^a^** | 98% | 97% | 102% | **72%** | 91% | -- |
|  | Ethylbenzene | -- | 104% **^a^** | 97% | 98% | 103% **^a^** | 90% ^a^ | 95% | 95% | 109% | **50%** | 95% | -- |
|  | n-Butylbenzene | -- | 95% **^a^** | 98% | 98% | 100% **^a^** | 96% ^a^ | 97% | 97% | 101% | 79% | 102% | -- |
|  | n-Decane | -- | 99% **^a^** | 83% | 117% | 128% **^a^** | 77% ^a^ | **133%** | 94% | 101% | 82% | 73% | -- |
|  | n-Dodecane | -- | 89% **^a^** | 96% | 93% | 98% **^a^** | 91% ^a^ | 95% | 98% | 93% | **68%** | 93% | -- |
|  | n-Nonane | -- | 99% **^a^** | 95% | 91% | 99% **^a^** | 89% ^a^ | 89% | 93% | 89% | **59%** | 89% | -- |
|  | n-Octane | -- | 108% **^a^** | 94% | 97% | 103% **^a^** | 89% ^a^ | 93% | 87% | 101% | **45%** | 88% | -- |
|  | n-Pentadecane | -- | 76% **^a^** | 91% | 97% | 116% **^a^** | 89% ^a^ | 129% | 88% | 123% | 78% | 91% | -- |
|  | n-Propylbenzene | -- | 100% **^a^** | 82% | 90% | 111% **^a^** | **77% ^a^** | 88% | 95% | 91% | **68%** | 91% | -- |
|  | n-Tetradecane | -- | 104% **^a^** | 92% | 100% | 107% **^a^** | 91% ^a^ | 105% | 97% | 104% | 80% | 91% | -- |
|  | n-Undecane | -- | 91% **^a^** | 98% | 94% | 92% **^a^** | 99% ^a^ | 97% | 96% | 93% | **67%** | 96% | -- |
|  | o-Dichlorobenzene | -- | 97% **^a^** | 98% | 92% | 118% **^a^** | 95% ^a^ | 93% | 96% | 99% | **68%** | 102% | -- |
|  | o-Xylene | -- | 103% **^a^** | 98% | 101% | 103% **^a^** | 91% ^a^ | 96% | 97% | 107% | **61%** | 91% | -- |
|  | p-Dichlorobenzene | -- | 103% **^a^** | 95% | 93% | **130% ^a^** | 88% ^a^ | 98% | 95% | 92% | **57%** | 88% | -- |
|  | p-Isopropyltoluene | -- | 105% **^a^** | 82% | 88% | 119% **^a^** | **79% ^a^** | 84% | 95% | 86% | **79%** | 88% | -- |
|  | sec-Butylbenzene | -- | 108% **^a^** | 83% | 93% | 121% **^a^** | **79% ^a^** | 88% | 96% | 92% | 83% | 90% | -- |
|  | Styrene | -- | 98% **^a^** | 94% | 92% | 101% **^a^** | **88% ^a^** | 94% | 89% | 99% | **38%** | 89% | -- |
|  | tert-Butylbenzene | -- | 120% **^a^** | 83% | 100% | **133% ^a^** | **78% ^a^** | 92% | 91% | 100% | 78% | 87% | -- |
|  | Toluene | -- | **149% ^a^** | 83% | 126% | 133% **^a^** | 86% ^a^ | 114% | 91% | **154%** | **20%** | 107% | -- |
|  | Xylenes (m and p) | -- | 102% **^a^** | 94% | 96% | 101% **^a^** | 86% ^a^ | 93% | 93% | 105% | **44%** | 90% | -- |

^a^ n=3

**References**

1. Anderson KA, Szelewski MJ, Wilson G, Quimby BD, Hoffman PD. Modified ion source triple quadrupole mass spectrometer gas chromatograph for polycyclic aromatic hydrocarbon analyses. J Chromatogr A. 2015; 1419: 89-98.

2. Kile ML, Scott RP, O'Connell SG, Lipscomb S, MacDonald M, McClelland M, et al. Using silicone wristbands to evaluate preschool children's exposure to flame retardants. Environ Res. 2016; 147: 365-72.

3. Donald CE, Scott RP, Blaustein KL, Halbleib ML, Sarr M, Jepson PC, et al. Silicone wristbands detect individuals' pesticide exposures in West Africa. Open Science. 2016; 3(8): 160433.

4. Parliament TE. Directive 2004/42/CE of the European Parliament and of the Council. In: Office EUP, editor. EUR-Lex. 2004.

5. Donald CE, Elie MR, Smith BW, Hoffman PD, Anderson KA. Transport stability of pesticides and PAHs sequestered in polyethylene passive sampling devices. Environ Sci Pollut R. 2016; 23: 12392-9.
